# A Bibliometric and Visualized Overview for the Evolution of Process Safety and Environmental Protection

**DOI:** 10.3390/ijerph18115985

**Published:** 2021-06-02

**Authors:** Jie Xue, Genserik Reniers, Jie Li, Ming Yang, Chaozhong Wu, P.H.A.J.M. van Gelder

**Affiliations:** 1 Safety and Security Science Group (S3G), Faculty of Technology, Policy and Management, Delft University of Technology, 2628BX Delft, The Netherlands; g.l.l.m.e.reniers@tudelft.nl (G.R.); m.yang-1@tudelft.nl (M.Y.); P.H.A.J.M.vanGelder@tudelft.nl (P.H.A.J.M.v.G.); 2 Intelligent Transportation Systems Center (ITSC), Wuhan University of Technology, Wuhan 430063, China; wucz@whut.edu.cn; 3 National Engineering Research Center for Water Transport Safety (WTSC), Wuhan University of Technology, Wuhan 430063, China; 4 Antwerp Research Group on Safety and Security (ARGoSS), Faculty of Applied Economics, University Antwerp, 2000 Antwerp, Belgium; 5 CEDON (Center for Economics and Corporate Sustainability), KU Leuven, Campus Brussels, 1000 Brussels, Belgium; 6 College of Safety Science and Engineering, Liaoning Technical University, Huludao 125105, China; 7 State Key Laboratory of Explosion Science and Technology, Beijing Institute of Technology, Beijing 100081, China

**Keywords:** bibliometrics, environmental protection, scientometric mapping, VOSviewer, Web of Science, evolutionary trends

## Abstract

This paper presents a bibliometric overview of the publications in the principal international journal Process Safety and Environmental Protection (PSEP) from 1990 to 2020 retrieved in the Web of Science (WoS) database to explore the evolution in safety and environmental engineering design and practice, as well as experimental or theoretical innovative research. Therefore, based on the WoS database and the visualization of similarities (VOS) viewer software, the bibliometric analysis and scientometric mapping of the literature have been performed from the perspectives of document types, publication and citation distribution over time, leading authors, countries (regions), institutions, the corresponding collaboration networks, most cited publications and references, focused research fields and topics, research trend evolution over time, etc. The paper provides a comprehensive and quantitative overview and significant picture representation for the journal’s leading and evolutionary trends by employing specific aforementioned bibliometric analysis factors. In addition, by reviewing the evolutionary trends of the journal and the proposed investigated factors, such as the influential works, main research topics, and the research frontiers, this paper reveals the scientific literature production’s main research objectives and directions that could be addressed and explored in future studies.

## 1. Introduction

Bibliometrics originated from library and information science [1]. A bibliographic analysis is mainly applied to characterize the structure and research trends of a specific field or journal by utilizing a quantitative methodology [2,3,4]. Additionally, it is a comprehensive visual analysis method augmented with network topology that could detect the influential authors, institutions, and countries in a specific research domain [5] and demonstrate a journal’s influence and productivity [1].

Moreover, scientific literature mapping by utilizing bibliometric methods is an effective complement to the traditional structured literature review, as it is able to provide a broader spectrum of research analysis [6,7]. Compared with a structured review, a bibliographic analysis provides a more wide angle on the analysis coverage breadth and the literature review depth [2]. In addition, bibliographic analysis has already been widely conducted in previous studies for analyzing various journals in different research subjects to explore and understand the specific research domain and research trends in the last few years. The typical bibliometrics analysis researches objects and topics, including journals, countries, authors, institutions, keywords, etc. Furthermore, the quantitative analysis is the fundament of bibliometric analysis, and the quantitative and qualitative are always combined during the practical analysis process. For instance, quantitative analysis is utilized with respect to the number of publications, while a qualitative analysis needs to be conducted when analyzing and summarizing a specific cluster’s theme. The quantitative analysis for the total number of citations of a particular publication could also reflect the quality and impact of the publication.

Many scholars who conducted the related research used bibliometric mapping methods. For instance, Li et al. [8] provided a bibliometric mapping review of the hotspots of lifecycle assessment for bioenergy. Zhi and Ji [9] explored the bibliometric mapping approach to give a review of quantitatively evaluated global scientific constructed wetlands research. Mao et al. [10] employed the bibliometric mapping to quantitatively analyze industrial wastewater treatment literature publications. Li et al. [11] did a preliminary overview of bibliometric mapping for the safety science community. Merigó, Miranda, Modak, Boustras, and de la Sotta [1] used bibliometric mapping to analyze forty years of safety science in terms of publications trends, leading producers (author, institutions, countries/regions), and highly cited papers and references also analyzed in the research. Additionally, as the knowledge carrier, scientific journals have published almost any research for a particular knowledge domain. The analysis of a specific journal helps understand the research of the area in some aspects. Several papers have conducted the research for journal analysis, e.g., *Journal of Infection and Public Health* [12], *Group Decision and Negotiation* [13], *Mechanism and Machine Theory* [14], *European Journal of Operational Research* [15], *Resources*, *Conservation and Recycling* [16], *Transportation Research Part A: Policy and Practice* [17], *Computers & Industrial Engineering* [18], *Knowledge-Based Systems* [19], *International Journal of Fuzzy Systems* [20], *Industrial Management & Data Systems* [21], etc.

Process Safety and Environmental Protection (PSEP) is the principal international journal covering the branches of engineering related to the research fields of safety of industrial processes and the protection of the environment. To explore the evolution in safety or environmental engineering design and practice, as well as experimental or theoretical innovative research, we strive to review the journal’s publication records and most significant trends through a general bibliometric analysis. Therefore, in the present study, the overview of the journal’s basic information and extraordinary contributions are recognized and analyzed in detail by the combination of qualitative and quantitative analysis, the related analyses involving the publication distribution and citation structure; leading authors, institutions, and countries (regions); influential publications; and focused research fields, as well as the research trend evolutionary process. Additionally, based on the information retrieved in the Web of Science Core (WoS) Collection database, the tool of the visualization of similarities (VOS) viewer, i.e., VOSviewer, which was developed by van Eck and Waltman [22], has been employed to perform a bibliometric analysis and scientometric mapping of publications from a visualization view.

The purpose of the present study is to (1) help related journal editors develop suitable strategies by examining more influential research types to achieve their development goals, (2) provide inspiration for academia and help them understand the most popular research fields and trends with the most publication potential, thus identifying and choosing the targeted research themes, and (3) concerning the benefits to readers, they can more intuitively and easily obtain more specific and accurate information that they are interested in from a large number of bibliometric data.

The remainder of this paper is organized as follows: first, Section 2 presents the materials and bibliometric analysis methods utilized in the paper. Second, the statistical analysis and graphical analysis results are detailed, including the publication trends and citation distribution; leading authors, institutions, and countries/regions; influential works in PSEP; and identified research fields and research evolutionary trends in the perspective of keyword co-occurrence. Additionally, the accompanying discussions are conducted in Section 3. Finally, Section 4 concludes the main findings of the paper and delivers the recommendations for the readers.

## 2. Materials and Methods

### 2.1. Bibliographic Data

In this paper, a typical journal PSEP with a high reputation in the research fields of safety of industrial processes and protection of the environment and with a relatively rapid increase of impact factor, quick review speed, and online article publication time, etc., was selected as the candidate journal to be analyzed.

**Figure 1:**
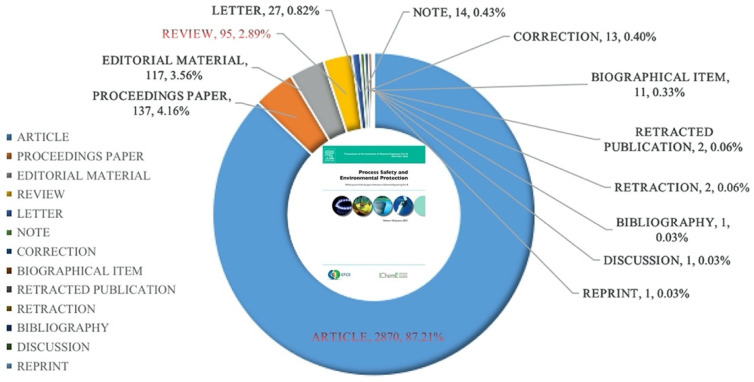
Document types of PSEP from 1990 to 2020.

The data were retrieved on 9 January 2021 from the WoS Core Collection, which is owned by Clarivate Analytics. The advanced search module was employed, and the strategy for obtaining data was, “Publication Name: SO = (Process Safety and Environmental Protection), Indexes = SCI-EXPANDED, Timespan = 1990–2020”. In total, 3152 publications were obtained from the Web of Science, and the PSEP publications had 13 different types (some of the papers were classified into more than one category). The proportion of each document type is shown in Figure 1. Note that articles and reviews are more essential document types in scientific outputs, and these two types have nearly 90.09% (2965 articles and reviews) in PSEP. The total number of citations was 44,879, and the average number of citations per publication was 14.24.

### 2.2. Bibliometric Methods and Analysis Tool

In the present paper, bibliometric methods were applied, and the bibliometric mapping tool VOSviewer was used to analyze the journal papers in a visual, user-friendly way. The bibliometric analysis originated from information and library science, which was first proposed by Otlet [23]. In the data science age, bibliometric methods were combined with network analysis and data visualization techniques, and then a new area named bibliometric mapping was produced. The bibliometric mapping was about quantitative methods (mathematics and statistics) for visually representing scientific literature based on bibliographic data.

Additionally, in bibliometrics, a threshold is used to select the minimum frequency of occurrence of the knowledge unit included in the network node. In the analysis of different knowledge units, the set of the specific threshold will have individual differences. Its primary purpose is to extract the core knowledge network formed by the analyzed knowledge units. It should be noted that the related results would be different by employing different thresholds in bibliometric analysis for different research topics and analytical problems, and there is generally no clear standard. In general, scholars set the threshold and conduct the related analysis directly under the premise that the problem explained is clear and the network constructed is easy to analyze and reasonable. The threshold usually is set to 3, 5, 10, 15, 20, 30, etc. [20,24,25,26], and the smaller the threshold setting, the larger and more complex the extracted knowledge network is.

Recently, bibliometric mapping analysis became popular not only inside the scientific communities of information and library science, but also in other scientific communities. More than 30 free tools have already been developed for bibliometric mapping, and VOSviewer is a famous tool among these tools [27,28]. VOSviewer is short for Visualization of Similarity, developed by van Eck and Waltman from Leiden University, the Netherlands, in 2010. The tool has several functions for bibliometric mapping, including collaboration analysis (e.g., authors, institutions, and countries/regions), topics analysis (e.g., keyword or terms), and citation-based analysis (e.g., bibliographic coupling and co-citations). Several papers have already applied VOSviewer to do bibliometric mapping analysis in environmental protection and safety-related topics, such as climate change [29], heavy metal removal [30], carbon emissions [31], contamination of water bodies [32], carbon capture and storage [33], soil remediation [34], safety culture [25], construction safety [35,36], process safety [37], domino effect [38], laboratory safety in universities [39], road safety research [40], etc.

## 3. Results and Discussion

### 3.1. Publication Trend and Citation Distribution

**Figure 2:**
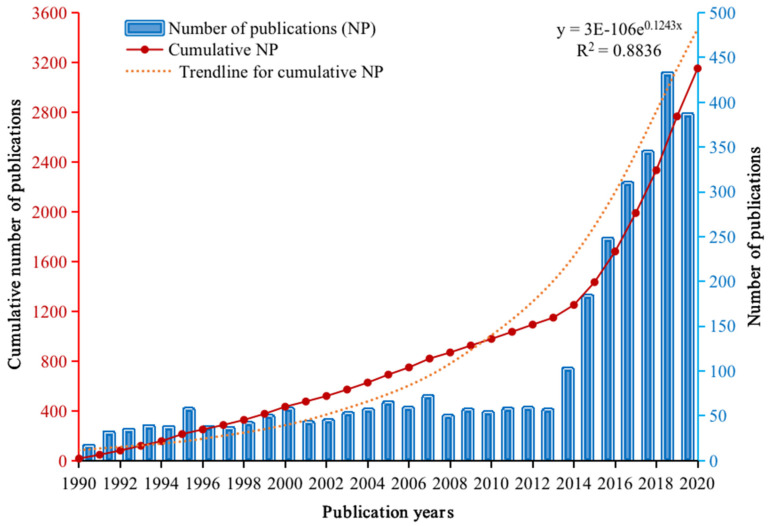
The number of publications in each year of PSEP from 1990 to 2020 in Web of Science.

The publication trend (including the number of papers) is the mirror and indicator for reflecting and measuring the scientific activities and attention to a specific domain. Figure 2 and Table 1 show the annual increase trend of PSEP publications. The increase of the annual outputs shows the increased attention to the topic scope of the PSEP from scientific communities. PSEP, as one of the leading journals in industrial process safety and environmental protection, has released a total of 16 publications in 1990, according to its earliest record in WoS. Moreover, the number of publications has increased slowly before 2013, and the average value for the number of publications per year before 2013 was around 48. After 2013, the publication trend increased rapidly, and the outputs reached more than 100 papers per year, with the number of papers in 2019 being 432 (exceeding 400 the first time). Additionally, the cumulative percentage of the number of publications showed that nearly 50% of cumulative publications from PSEP were published after 2016, which means that the most recent five years (2016–2020) have contributed roughly half of all of the papers that have been published in PSEP from 1990 to 2020.

### 3.2. Leading Authors, Institutions, and Countries/Regions

#### 3.2.1. Leading Authors and Collaborations

**Figure 3:**
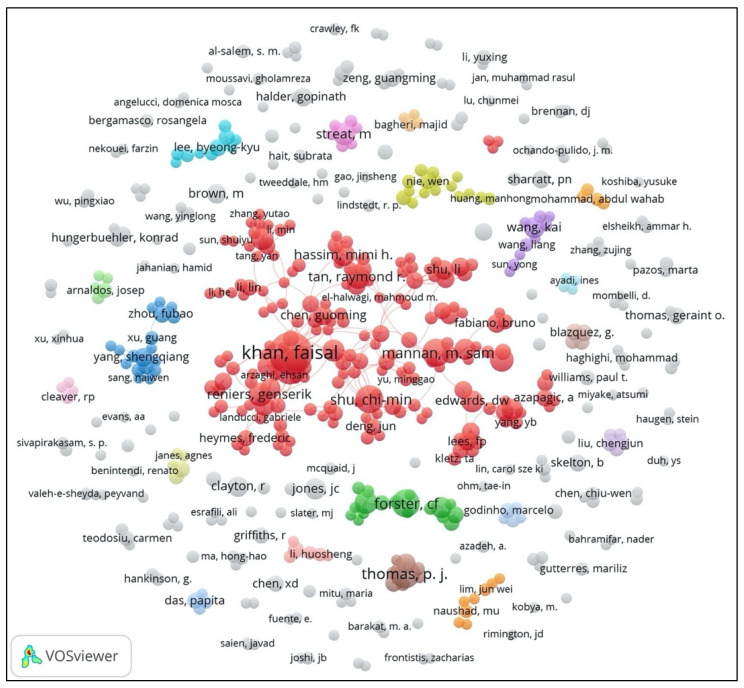
The whole authors’ collaboration network of PSEP from 1990 to 2020 (the minimum number of publications = 3).

Authors are the knowledge producers of PSEP, and an author’s production and collaboration analysis can easily show the leading researchers and the author’s social connectedness in PSEP. The whole author’s collaboration network is illustrated in Figure 3, and the author who had a minimum number of publications of 10 was regarded as the leading author in PSEP. Table 2 lists the leading authors of PSEP, and indicates that there are 69.70% (23/33) of them within the giant connected component (GCC) of the authors’ collaboration network (cluster 1, the red group (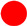) in Figure 3). Considering the sparsely connected network structure of other groups, only the GCC of the authors’ collaboration network was selected to analyze the author’s social connection in PSEP. Note that authors with only the publication type of editorial material are not included in Table 2. The node size is proportionate to the number of publications of an author; the node color represents the clusters of authors in the same group. Additionally, the links between authors present the collaboration relations between authors, and the wideness of the link shows the authors’ collaboration strength.

As shown in Table 2, Faisal Khan (Mem. Univ. Newfoundland, Canada) is the most productive author in PSEP with 62 publications. He is also the only author with more than 50 papers in PSEP, followed by Paul Amyotte (Dalhousie University, Canada), P.J. Thomas (University of Bristol, UK), C.F. Forster (University of Birmingham, UK), M. Sam Mannan (Texas A&M University, USA), and Chi-Min Shu (National Yunlin University of Science and Technology, Taiwan), with more than 20 papers published in PSEP, ranked in the top 2–6 positions, respectively. Furthermore, Kai Wang (China University of Mining & Technology, China) holds the largest average publication year (APY) of 2019.00, and Gordon Mckay (Hamad Bin Khalifa University, Qatar) is the author who has the most citations (2897) and the highest average number of citations (193.13). The productivity distribution of the (leading) authors was not balanced; there were just a few authors who have published a large number of papers, resulting in the uneven distribution of the total number of citations of leading authors. In addition, the highly productive editorial board members of PSEP are also highlighted in Table 2. Among the leading authors, 24.24% (8/33) are editorial board members, which indicates that the editorial board members play a relatively important role among the leading authors, as well as within the process safety and environmental protection research domain. Additionally, as shown in Table 2, 27.27% (9/33) of the leading authors originate from the United Kingdom.

#### 3.2.2. Leading Countries/Regions and Collaboration

**Figure 4:**
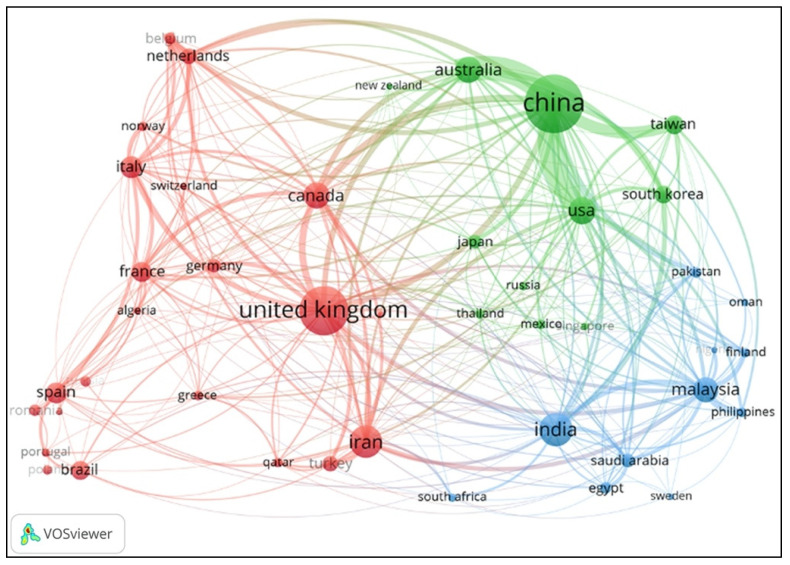
Countries/regions collaboration network in PSEP publications.

The countries/regions cooperation relation in the explored field was also visualized and analyzed by utilizing collaboration networks analysis to investigate affiliated countries and institutes through the VOSviewer software. As shown in Figure 4, the minimum number of publications was 10, and 44 countries/regions were included in the network. It should be noted that a node was apportioned to each co-author of a publication in the networks. The node’s color presents the average time for the publications of each country [41]. The node’s size denotes the related publication number, and the thickness of the links indicates the international collaboration degree [38,42], i.e., the larger the node is, the more critical the country/region is, and the thicker the line is, the closer the cooperation relationship between countries/regions.

Figure 4 indicates that China (with 678 publications and 9615 total citations) and the United Kingdom (with 476 publications and 7517 total citations) have been at the forefront and play the predominant roles in PSEP. Meanwhile, China mainly collaborates with the USA, Australia, Taiwan (region), United Kingdom, South Korea, Japan, Canada, Netherlands, etc. Similarly, the close collaboration countries/regions with the United Kingdom are Canada, China, Australia, Iran, Germany, India, Italy, Netherlands, France, etc. Additionally, among the close collaboration countries/regions, China and the USA have the most immediate cooperation and research relationship in PSEP. Moreover, as shown in Table 3, in terms of the publication time, Thailand (with the largest average publication year (APY) of 2018.14), Qatar (2017.62), and Tunisia (2017.43) are the top three relatively active research countries recently, and have the latest output in PSEP according to the investigated results from the core database of the WoS. Furthermore, the Philippines (with an average citation of 28.59), Finland (27.13), and Saudi Arabia (25.36) are the only three countries whose average citations exceeded 25. Generally, more international collaboration needs to be promoted and enhanced to share knowledge globally in the future.

#### 3.2.3. Leading Institutions and Collaborations

**Figure 5:**
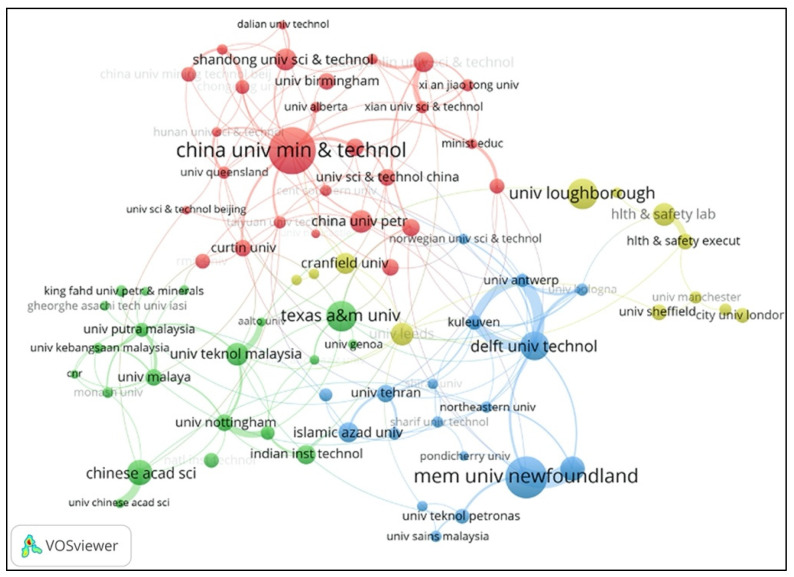
Institution collaboration network of PSEP publications from 1990 to 2020.

Figure 5 illustrates the institution collaboration network for PSEP during the entire explored timespan. The node’s size presents the number of publications (the bigger the note is, the more publications the institution has), and the links between two nodes indicate the collaboration relationship between two institutions (the thicker the link is, the closer the cooperation they have). In addition, each institution in the network has a minimum of 10 publications in PSEP, and 77 institutions meet the threshold. As shown in Table 4, China University of Mining & Technology (China) has the highest number of publications at 73; Nanjing Tech University (China) has the largest APY at 2018.87. In Canada, the Memorial University of Newfoundland gets the highest number of total citations of 1854 among the leading institutions of PSEP, and Dalhousie University holds the highest average citations of 37.24 (the only institution whose average numbers of citations exceeded 30). With a total number of citations of 1229, Dalhousie University is also the institution whose total number of citations exceeded 1000, except Memorial University of Newfoundland. Additionally, 29.63% (8/27) of the leading institutions are from the United Kingdom.

### 3.3. Influential Works

#### 3.3.1. Influential Works Published by PSEP

Publications with a large number of citations often indicate the influence of the publication in a specific research domain, i.e., the number of publications exceeding a certain citation threshold allows the identification of the number of publications that have a certain level of influence [43,44]. In this paper, the publications with more than 100 citations are identified as influential works in PSEP. Therefore, 25 publications are listed in Table 5. The paper by Ho and McKay [45] held the highest number of citations of 1530 and the biggest average number of citations per year of 66.52. Moreover, there were seven publications with a total number of citations of more than 200. Additionally, there were two papers among the first five most cited papers (2/5 = 40.00%), and 24.00% (6/25) of the top 25 most cited papers were review papers, while, as shown in Figure 1, only 2.89% of all publications were the review papers. The statistical fact that a relatively small number of publications accomplished with a relatively high total number of citations indicated that the document type of review paper was more likely to get more citations.

**Figure 6:**
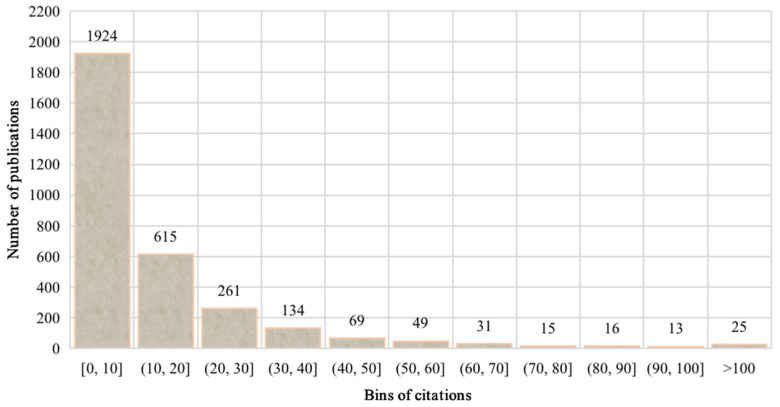
Citation distribution of PSEP publications.

Furthermore, Figure 6 demonstrates the citation distribution of PSEP publications from 1990 to 2020. Overall, according to the increasing of the number of citations, the number of publications gradually decreased. In addition, there were 1924 publications, the highest number of publications among various intervals, that had no more than ten citations. Note that, among the publications cited no more than ten times, there were 447 publications with no citations. Considering that a paper’s publication requires a certain period, the citations cannot be counted in time. However, except for the 183 publications of 2020, 59.06% (264/447) of the publications had zero citations. Note that, since most of the influential works’ research topics were cross-fused with the research hotspots in Section 3.4, the related literature productions are not discussed and analyzed in detail in this section.

.

#### 3.3.2. Influential Works Cited by PSEP

**Figure 7:**
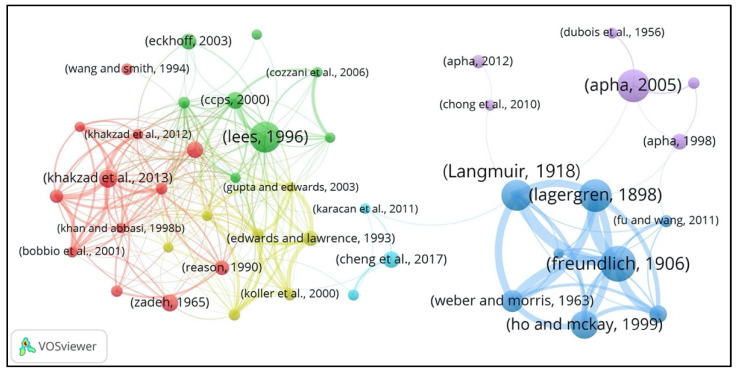
Co-citation network of highly cited reference groups based on co-citation strength.

Highly cited works cited by PSEP papers in our local dataset can be considered the intellectual bases of PSEP. The co-citation network of highly cited references (the minimum number of citations of a paper was 15) was constructed. In total, 43 highly cited references were identified and obtained from the 89,287 references of PSEP. The co-citation network among these 43 papers is displayed in Figure 7. The node stands for a highly cited reference, and the size is proportional to the number of cites from the PSEP papers. The label here just shows the first author or first two authors and the publication year of a paper. In addition, links between each node present the co-citation relations of highly cited references. Link wideness indicates the co-citation strength between these references. The color shows the different groups of these references, which was clustered based on the co-citation strength of these references by using the bibliometric data analysis based on the clustering method included in the widely used VOSviewer software, as introduced in Section 2.2. Note that the investigated references can only be included in one cluster, and their position in the overall network and the connections to the references in other clusters show how closely related it is, both within its own cluster and with other clusters.

Furthermore, Table 6 lists the highly cited references of PSEP publications ranked by the number of citations. Most of the influential works cited by PSEP were journal articles, accounting for 81.40% (35/43). Additionally, the blue group (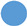) was the biggest cluster with the most citations (277), and the red group (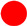) was the biggest cluster with the most publications (12). As shown in Figure 7, there were six clusters (groups) for the highly cited references:

The blue group (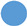) was primarily concentrated on environmental protection theories and techniques (especially adsorption theory and application). The most influential works in this group were the theory for adsorption in solution [46,47,48] and adsorption of gases [49], modeling for the sorption processes [50], and isotherms systems [51]. Moreover, the review on methodologies and techniques for removing heavy metal ions from wastewaters by Fu and Wang [52] and the fundamental theory for the constitution and properties of solids and liquids by Langmuir [53], etc., are also impactful works in this group.

The red group (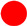) mainly focused on the methodologies and models for process safety and risk management in chemical and process industries. Dynamic safety analysis and risk assessment theory and models, e.g., Bayesian theory [54,55], bow-tie approach [56], etc., are developed and widely used, and the aforementioned research was recognized as the influential works. Additionally, the other impactful original research was the reviews of the available techniques and methodologies for risk analysis in chemical process industries by Khan and Abbasi [57] and the methods and models in process safety and risk management by Khan et al. [58], the research for fuzzy sets by Zadeh [59], the predictive accident model for system hazard identification, prediction, and prevention by Rathnayaka et al. [60], and the utilization of Bayesian network and fault tree approaches for the safety analysis in process facilities [61] and dependable systems [62], etc.

The yellow group (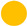) mainly focused on the inherent safety and hazard identification and assessment in chemical and process industries. The group contained the early influential works by Edwards and Lawrence [63] on the exploration for the relation between plant costs and the inherent safety of chemical process routes, and the multivariate system hazard identification and ranking methods for fire and explosion and toxic chemical release hazards by Khan and Abbasi [64]. In addition, Gupta and Edwards [65] proposed a graphical methodology for inherent safety measurement, Khan and Amyotte [66] detailed the cost and system design model for integrated inherent safety index, Koller et al. [67] presented a safety, health, and environmental impact assessment methodology for selecting the most reliable data from a variety of substance databases or estimation method, and Khan et al. [68] developed a safety-weighted hazard index for chemical process industry hazard identification and risk assessment. The research mentioned above was widely cited by the publications in PSEP and identified as influential works as well.

The green group (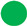) concentrated on loss prevention in process industries. The influential works were primarily focused on major accident hazards, accident causes, and consequences analysis, and some specific original contributions on managing the risk of the domino effect of chemical accidents. The most cited works in this group were the book on loss prevention in the process industry by Lees [69], the book on chemical process safety by Crowl and Louvar [70], the book on the guidelines for chemical process quantitative risk analysis by CCPS [71], and the book on the identification, assessment, and prevention of hazards for dust explosion accidents, which presented the evaluation of prevalent activities, testing methods, and design measures for safe operation techniques by Eckhoff [72]. The significant and influential articles included the research for the domino effect for chemical accident features, sequences analysis, and accidental event escalation threshold assessment, as well as the research by Khan and Abbasi [73] on the common causes or errors and consequences of major accidents in chemical process industries, etc.

The purple group (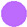) was primarily about waste treatment (especially wastewater treatment). The most significant original research contributions in this group concerned the work on the examination of water and wastewater standard methods by APHA [74,75,76], and the significant overview or review articles included the analyzing and summarizing of the recent developments for the technologies of photocatalytic water treatment [77] and landfill leachate treatment [78], etc.

The azure group (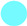) mainly focused on coal mine safety; it primarily contained compendia works, for instance, Cheng et al. [79] designed an intelligent gel for fire prevention and extinguishing to control coal spontaneous combustion (CSC), Wang et al. [80] established a model of airflow dust migration for underground mine tunnels and achieved a better air curtain dust suppression effect, and Karacan et al. [81] delivered an overview for the capture and utilization of coal mine methane in the perspective of mining safety and greenhouse gas reduction, etc. The aforementioned works were highly influential in this group.

### 3.4. Research Fields Identification and Research Trends Evolution

Keywords are one of the essential elements supplied by the authors of the paper to show the paper’s core content. Author keywords are imperative, since they are used as the topics/concepts/methods that are presented to deliver and communicate to the scientific community by the authors. The author keyword co-occurrences network demonstrates another perspective of themes in PSEP, and it can be observed that it illustrates the main author keywords that frequently occur together in PSEP.

**Figure 8:**
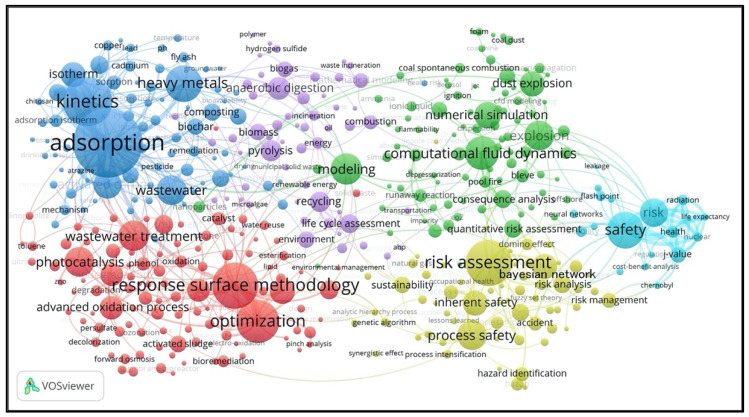
Keyword co-occurrence cluster of PSEP papers.

Considering the fact that many keywords only appeared a few times, they obviously have not had significant influences on the main themes of PSEP. Therefore, in the present study, to focus on the main themes, only the keywords occurring at least five times were selected to construct the co-occurrence analysis map and indicate the research topics. Thus, 407 keywords were extracted based on the threshold of the keyword’s frequencies. The keyword co-occurrence network for the clusters (groups) of PSEP is shown in Figure 8. Note that the larger the nodes and character fonts, the more often the keywords are used.

Obviously, the keyword “adsorption” is the most used author’s keyword with 122 occurrences, followed by “kinetics” (80), “response surface methodology” (74), “risk assessment” (73), “optimization” (67), “heavy metals” (56), “safety” (56), etc. There were six clusters of keywords, separated by different colors and representing the following themes:

The blue group (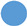) was mainly concentrated on waste and pollutants remediation. This cluster included keywords such as “adsorption” (122), “kinetics” (80), “heavy metals” (56), “wastewater” (43), “activated carbon” (40), “isotherm” (34), “biosorption” (23), “composting” (22), “thermodynamic” (22), “volatile organic compound” (22), etc.

The red group (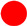) mainly focused on environmental protection methodologies and technologies. This cluster contained keywords such as “response surface methodology” (74), “optimization” (67), “wastewater treatment” (44), “photocatalysis” (43), “biodiesel” (34), “advanced oxidation process” (32), “electrocoagulation” (31), “biodegradation” (30), “artificial neural network” (24), “water treatment” (24), etc.

The purple group (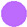) was mainly about waste management and sustainable development. This cluster was represented by keywords such as “anaerobic digestion” (31), “pyrolysis” (28), “recycling” (27), “lifecycle assessment” (24), “biomass” (21), “environment” (20), “biogas” (19), “mathematical modeling” (18), “combustion” (17), “mass transfer” (15), “energy” (14), “sustainable development” (12), “emissions” (12), etc.

The green group (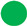) mainly concentrated on accident prediction and hazard assessment methods and models (especially fire and explosion mitigation). This cluster was characterized by keywords such as “computational fluid dynamics” (49), “modeling” (46), “explosion” (43), “numerical simulation” (37), “dust explosion” (34), “consequence analysis” (23), “gas explosion” (19), “hydrogen” (19), etc.

The yellow group (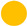) mainly focused on the process safety and risk assessment in chemical and process industries. This cluster was represented by keywords such as “risk assessment” (73), “process safety” (43), “Bayesian network” (34), “inherent safety” (34), “risk analysis” (23), “human factors” (22), “risk management” (18), “hazard identification” (17), “process design” (17), etc.

The azure group (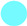) mainly focused on safety and risk management strategy and cost–benefit analysis. This cluster was composed of keywords such as “safety” (56), “risk” (40), “J-value” (19), “health” (14), “reliability” (13), “flash point” (11), “radiation” (11), “nuclear” (10), etc.

**Figure 9:**
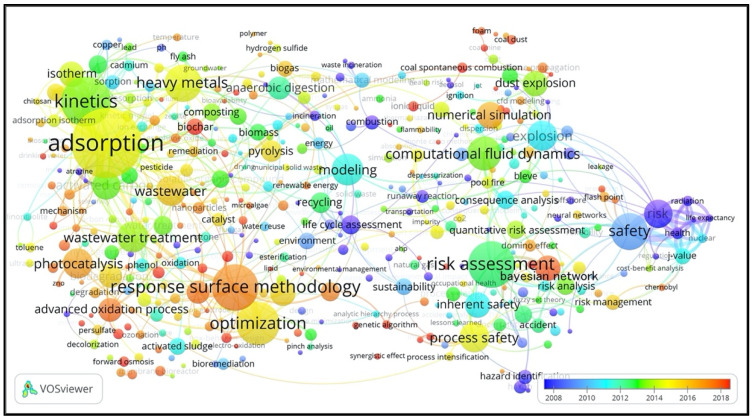
The evolution of PSEP research keywords over time based on the average publication year.

Additionally, to further understand and analyze the research topics, evolutionary process, and research frontiers for PSEP, the chronological evolution of research keywords based on the average publication date for the publications in which the keywords appeared in PSEP is demonstrated in Figure 9. Note that the VOSviewer software automatically settled the most suitable time interval and span for 407 keywords based on the publication time (i.e., average publication year) of each keyword. The main research topics in various periods could be recognized and summarized based on the occurrence frequency of the keywords in each period. Table 7 summarizes the top 20 keywords that appeared most frequently in each sub-period.

Overall, the PSEP was mainly focused on the topics related to the fields of safety of industrial processes and protection of the environment. In the present study, to explore and extract the evolutionary process and trends of the main research topics in PSEP, the special topics of publications in the separated four periods are summarized and analyzed.

Earlier publications (before 2008) were focused mainly on the topics concerned with waste incinerator design and combustion efficiency analysis [89,90,91,92], treatment and utilization of (municipal) solid waste [93,94,95], incineration residues and emissions management [96,97,98], economic and environmental impact assessments and sustainable development [99,100,101,102], reduction of flue gas emissions from fuel combustion [103], risk analysis for offshore platform-related problems [104,105], boiling liquid expanding vapor explosion (BLEVE) (especially related to liquefied petroleum gas (LPG)) [106,107], risk-based decision-making [108,109], human health risk analysis [110], thermal radiation safety assessment [111], runaway reaction inhibition system design and evaluation [112], loss prevention analysis in process industry [113], etc. The main analysis/process methods include incineration [114], biological phosphorus removal [115], catalytic combustion [116], lifecycle assessment (LCA) [117,118], hazard and operability (HAZOP) analysis [119,120], mathematical modeling [89], fuzzy logic theory [121,122], computer-aided fault tree analysis (FTA) [109], etc.

Between 2008 and 2012, publications were devoted to the research topics about the application and optimization of anaerobic digestion (especially the anaerobic digestion of organic solid wastes and wastewater sludges) [123,124], resource recycling and waste regeneration reuse [125,126], activated sludge utilization and treatment [127,128,129], biomass-derived liquid biofuels [130], optimal process design (especially based on inherent safety or under uncertainty) [131,132,133], explosion overpressures analysis and prediction (especially caused by a gas explosion) [134], explosion accident hazard assessments and prevention (especially coal combustion/gas explosion) [135], inherent safety and cost evaluation [136,137], human factors in safety management [138], J-value safety analysis [139,140], etc. Additionally, anaerobic digestion [124,141], biosorption [142], biomass gasification [143], vacuum thermal recycling [144], computational fluid dynamics (CFD) [145], principal component analysis (PCA) [132], analytic hierarchy process (AHP) [146], Monte Carlo simulation (MCS) [147], etc., became the widely used techniques/methods among scholars during this period.

Subsequently, during the period between 2013 and 2017, the research emphasis turned to topics such as pollution management framework [148], analysis of isotherms and kinetics of adsorption [149], heavy metals risk assessment and removal [150], wastewater treatment (especially based on the advanced oxidation process (AOP) and electrocoagulation (EC)) [151], biodiesel processing and production [152], process safety framework and model [153], risk-based design [154], hazardous materials transportation safety [155,156], investigation and risk analysis of fire and explosion accidents (especially dust explosion) [154,157], etc. In terms of analysis/process methods, AOP [158], EC [159], photocatalysis degradation [160], activated carbon adsorption [161], biodegradation [162], catalytic pyrolysis [163], response surface methodology (RSM) [164], quantitative risk assessment (QRA) [165,166], multi-objective optimization [167], fuzzy FTA [168], numerical simulation [169,170], and Bayesian network (BN) [154], etc., were widely applied by scholars.

In recent years (2018 and beyond), some of the focused research topics/methods were the continuation of the previously initiated research areas, e.g., BLEVE, HAZOP, AOP, EC, CFD, RSM, MCS, QRA, BN, PCA, etc. In addition, a few new research topics emerge in this period, such as catalytic degradation of organics (especially utilizing a microbial community) [171,172], application of ionic liquids [173], degradation and toxicity analysis [174,175], the application of microwave for waste treatment [176,177], safety of drinking water and its treatment [178], mineralization technology for pollutant emission control [179], Industry 4.0 [180,181,182], mine fires and CSC prevention [183,184], air leakage measurement and sealing techniques [185,186], risk analysis and prevention considering synergistic effects and domino effects [187,188], etc. It is obvious that scholars pay more attention to advanced techniques, as well as traditional methods, to conduct real-world industrial problems, for instance, catalytic ozonation [189], forward osmosis [190], photocatalytic [191], electro-oxidation [191], biochar adsorption [192], artificial neural networks [193], genetic algorithm [194], (global) sensitivity analyses [195,196], process optimization method (e.g., RSM, CCD) [197,198], bowtie analysis [199], fuzzy AHP [200,201], dynamic BN [187,202], etc., for environment protection and process safety and risk analysis.

## 4. Conclusions

This study presents a bibliometric journal analysis perspective to explore the evolution in safety or environmental engineering design and practice, as well as experimental or theoretical innovative research. Various bibliometric analyses, scientometric mapping, and statistical techniques are utilized to identify and unearth the evolution trends and detailed characteristics of the publications as indexed by the Web of Science. To validate the application of our purpose, the principal and influential international journal PSEP is taken as the case study journal to explore the influencing factors behind the rapid development of journals and reveal the main research trends of the safety of industrial processes and the protection of the environment.

The main findings for our case study journal could be summarized as follows: (a) until the statistical time of this paper, PSEP has published 3152 literature productions and drawn 44,879 citations, and the corresponding number keeps increasing over time. (b) Faisal Khan is the most productive author and the only author with more than 50 papers in PSEP, followed by Paul Amyotte and P. J. Thomas. Additionally, Gordon Mckay is the most influential author (with 2897 citations and the highest average number of citations of 193.13). Moreover, the editorial board members play a relatively important role among the leading authors and within the process safety and environmental protection research domain. (c) China is the leading country with the highest number of publications and citations, followed by the United Kingdom and India. Additionally, China and the USA have the closest cooperation and research relationship in PSEP. It is expected that more international collaboration could be promoted and enhanced to share knowledge globally in the future. (d) The leading institutions are China University of Mining & Technology (with the highest number of publications of 73), Nanjing University of Technology (with the largest average publication year of 2018.87), Memorial University of Newfoundland (with the highest number of total citations of 1854), and Dalhousie University (with the highest average citations of 37.24). Furthermore, 29.63% of the leading institutions are from the United Kingdom, and the most productive countries and institutions both regard China. (e) The most influential work published in PSEP held 1530 citations and the biggest average citations per year of 66.52. In addition, there are seven literature productions with the minimum citations of 200, and the document type of review paper is more likely to get more citations. However, 61.04% publications in PSEP have no more than ten citations, and there are still a certain number of publications that are not cited. Furthermore, there are 43 highly cited references (most of them are journal articles) that can be regarded as the core intellectual bases of PSEP. These works concern adsorption theory, process safety and risk management methods and models, inherent safety, loss prevention, domino effect, waste treatment, and coal mine safety. The related reviews for the highly cited references could deliver further detailed insights in the explored domain for scholars. (f) Several keywords/topics, such as “adsorption”, “kinetics”, “response surface methodology”, “risk assessment”, “optimization”, “heavy metals”, and “safety”, are the most popular hotspots of PSEP. Additionally, the popular keywords in various periods reveal the chronological evolutionary process and trends of the emerging or long-lasting research hotspots of PSEP during the last 30 years.

The topics of the selected publications from the case study journal mainly concentrate on the research fields of protection of the environment and safety of industrial processes. For specific research areas, the mainstream research areas of our case study journal, including the waste and pollutants remediation, environmental protection methodologies and technologies, waste management and sustainable development, accident prediction and hazard assessment methods and models, process safety and risk assessment, and safety and risk management strategy and cost–benefit analysis, etc., are recognized. In addition, some new emerging topics in the research community could be highlighted and recommended for scholars and stakeholders. In respect to environmental protection, some emerging topics are recognized, such as catalytic pyrolysis of waste, sustainability of the production of fuels, analysis of kinetics and adsorption mechanisms, activated carbon adsorption, supercritical water processes, climate change mitigation technology (especially catalytic conversions of greenhouse gas), catalytic degradation of organics (especially utilizing microbial community), application of microwave for waste treatment, safety of drinking water and its treatment, mineralization technology for pollutant emission control, etc. In view of industrial process safety, the mainly focused topics are failure of complex systems, mine fires and coal spontaneous combustion prevention, inherent safety and cost evaluation, risk analysis and prevention considering synergistic effects and domino effects, optimization for hazardous materials transportation, J-value analysis and assessment of accidents, safety culture and process safety education, human and organizational factors of safety, etc. As for Industry 4.0 (especially smart manufacturing and production, big data, Internet of Things), accompanied with artificial intelligence (e.g., the application of artificial intelligence for wastewater treatment and deep learning-based forecast modeling for safety/risk-related topics), are widely used as the hotspots for the research fields of PSEP. In terms of techniques/methods, heterogeneous catalysis, photocatalysis degradation, advanced oxidation process, biochar for adsorption, machine learning (especially deep learning), response surface methodology, numerical simulation, HAZOP, bowtie, dynamic Bayesian network, dynamic and computer-aided fault tree analysis, hybrid artificial neural network, genetic algorithm approach, etc., are widely used and applied advanced emerging techniques, as well as traditional methods, among global scholars for solving the environment protection, safety, and risk analysis problems. The aforementioned new future trends contribute to offering safer and cleaner living and production environments for human beings, which will further promote the harmonious coexistence of man and nature and achieve social and economic development sustainability.

This paper provides a comprehensive and quantitative overview and significant picture representation for the journal’s leading and evolutionary trends by employing specific bibliometric key impact factors, such as the document types, publication distribution and citation structure, most cited works, the trends of research topics, and prominent contributing authors, countries (regions), and institutions, etc. Additionally, by reviewing the evolution trends of the journal and the proposed investigated factors, such as the influential works, main research topics, and the research frontiers, this paper delivers various research objectives and directions that could be addressed and explored in future studies for related scholars worldwide, as well as for related journal editors, to position their journal to align with the focus topic of the safety of industrial processes and the protection of the environment. However, note that the results obtained from the present paper are dynamic, and may change over time along with the emergence of new research hotspots or mainstream subjects and some specific variables increasing/decreasing in the study.

In the follow-up research, some other data sources, e.g., Scopus, Google Scholar, and EconLit, etc., and some other document types, e.g., books, proceedings books, PhD theses, etc., could be contained as a supplement for scholars applying our proposed methodology for exploring the evolutionary trends for a particular knowledge domain. Additionally, some comparative analysis and inductive research for different journals in a specific research area could be conducted in further study. Nevertheless, some expert knowledge should be considered when selecting the most relevant journals and articles, especially for a very wide study field that may lead to a huge workload of a subsequent analysis and an overloading and time-consuming phenomena for the analysis tools.

## Figures and Tables

**Table 1:** Annual publications and citations of PSEP.

Years	NP	% of 3152	CNP	% of CNP	TC	CPP
1990	16	0.51%	16	0.51%	14	0.88
1991	31	0.98%	47	1.49%	186	6.00
1992	34	1.08%	81	2.57%	204	6.00
1993	38	1.21%	119	3.78%	275	7.24
1994	37	1.17%	156	4.95%	249	6.73
1995	57	1.81%	213	6.76%	219	3.84
1996	37	1.17%	250	7.93%	276	7.46
1997	36	1.14%	286	9.07%	560	15.56
1998	41	1.30%	327	10.37%	3266	79.66
1999	49	1.55%	376	11.93%	519	10.59
2000	57	1.81%	433	13.74%	1028	18.04
2001	42	1.33%	475	15.07%	656	15.62
2002	44	1.40%	519	16.47%	353	8.02
2003	52	1.65%	571	18.12%	957	18.40
2004	56	1.78%	627	19.89%	785	14.02
2005	64	2.03%	691	21.92%	1033	16.14
2006	58	1.84%	749	23.76%	1194	20.59
2007	71	2.25%	820	26.02%	1660	23.38
2008	49	1.55%	869	27.57%	1516	30.94
2009	56	1.78%	925	29.35%	1026	18.32
2010	53	1.68%	978	31.03%	1360	25.66
2011	57	1.81%	1035	32.84%	1720	30.18
2012	58	1.84%	1093	34.68%	1527	26.33
2013	56	1.78%	1149	36.45%	1297	23.16
2014	102	3.24%	1251	39.69%	2005	19.66
2015	183	5.81%	1434	45.49%	3863	21.11
2016	247	7.84%	1681	53.33%	4508	18.25
2017	309	9.80%	1990	63.13%	5062	16.38
2018	344	10.91%	2334	74.05%	4275	12.43
2019	432	13.71%	2766	87.75%	2742	6.35
2020	386	12.25%	3152	100.00%	544	1.41

Note: NP = number of publications, CNP = cumulative number of publications, TC = total citations, CPP = citations per paper = TC/NP. The colors range from green to red in the related column indicate the smaller the number and the closer the color is to green in that column.

**Table 2:** Leading authors in PSEP based on the number of publications (minimum number of publications = 10).

Rank	Author	EBMs	C/R	Institution	NP	TC	APY	CPP
1	Khan, Faisal	Y	Canada	Mem. Univ. Newfoundland	62	1938	2014.50	31.26
2	Amyotte, Paul	N	Canada	Dalhousie Univ.	28	1101	2011.96	39.32
3	Thomas, P. J.	Y	UK	Univ. Bristol	27	251	2012.48	9.30
4	Forster, CF	N	UK	Univ. Birmingham	24	243	1996.17	10.13
5	Mannan, M. Sam	N	USA	Texas A&M Univ.	24	346	2012.13	14.42
6	Shu, Chi-Min	N	Taiwan	Natl. Yunlin Univ. Sci. & Technol.	23	172	2015.26	7.48
7	Tan, Raymond R.	N	Philippines	De La Salle Univ.	17	417	2014.12	24.53
8	Reniers, Genserik	N	Belgium	Univ. Antwerp/Delft Univ. Technol.	16	137	2018.25	8.56
9	Richardson, SM	N	UK	Imperial Coll. London	16	97	1996.00	6.06
10	Hassim, Mimi H.	Y	Malaysia	Univ. Teknol. Malaysia	15	197	2015.00	13.13
11	Mckay, Gordon	N	Qatar	Hamad Bin Khalifa Univ.	15	2897	2003.00	193.13
12	Streat, M	N	UK	Univ. Loughborough	15	443	2001.47	29.53
13	Yang, Ming	N	Netherlands	Delft Univ. Technol.	15	250	2016.07	16.67
14	Edwards, DW	Y	UK	Univ. Loughborough	14	307	2000.71	21.93
15	Jiang, Juncheng	N	China	Changzhou Univ.	14	51	2018.79	3.64
16	Wang, Deming	N	China	China Univ. Min. & Technol.	14	240	2017.93	17.14
17	Abbassi, Rouzbeh	Y	Australia	Macquarie Univ.	13	106	2017.69	8.15
18	Jones, JC	N	UK	Univ. Aberdeen	13	19	2005.85	1.46
19	Pasman, Hans J.	N	USA	Texas A&M Univ.	13	249	2012.15	19.15
20	Stephenson, T	N	UK	Cranfield Univ.	13	189	1999.77	14.54
21	Swithenbank, J	N	UK	Univ. Sheffield	13	163	2001.62	12.54
22	Wang, Kai	N	China	China Univ. Min. & Technol.	13	76	2019.00	5.85
23	Cozzani, Valerio	N	Italy	Univ. Bologna	12	139	2016.42	11.58
24	Khakzad, Nima	N	Canada	Ryerson Univ.	12	376	2017.58	31.33
25	Chen, Guoming	N	China	China Univ. Petr.	11	152	2017.82	13.82
26	Jones, R. D.	N	UK	City Univ. London	11	106	2009.73	9.64
27	Shu, Li	N	Australia	RMIT Univ.	11	111	2017.18	10.09
28	Fabiano, Bruno	Y	Italy	Univ. Genoa	10	271	2013.80	27.10
29	Foo, Dominic C. Y.	Y	Malaysia	Univ. Nottingham	10	136	2015.10	13.60
30	Halder, Gopinath	N	India	Natl. Inst. Technol. Durgapur	10	76	2018.50	7.60
31	Shon, Ho Kyong	Y	Australia	Univ. Technol. Sydney	10	60	2017.80	6.00
32	Yang, Shengqiang	N	China	China Univ. Min. & Technol.	10	67	2018.90	6.70
33	Zhang, Laibin	N	China	China Univ. Petr.	10	128	2016.60	12.80

**Table 3:** Leading countries/regions in PSEP based on the number of publications (minimum number of publications = 20).

Rank	C/R	Continent	NP	TC	APY	CPP
1	China	Asia	678	9615	2017.27	14.18
2	UK	Europe	476	7517	2006.32	15.79
3	India	Asia	240	4251	2015.92	17.71
4	Iran	Asia	228	3514	2017.00	15.41
5	USA	North America	160	2357	2013.86	14.73
6	Australia	Oceania	152	1739	2013.55	11.44
7	Canada	North America	147	2957	2013.82	20.12
8	Malaysia	Asia	136	3181	2016.00	23.39
9	Italy	Europe	113	1431	2014.58	12.66
10	Spain	Europe	102	1227	2015.61	12.03
11	France	Europe	91	1285	2012.60	14.12
12	Brazil	South America	82	784	2016.99	9.56
13	Taiwan	Asia	82	865	2015.09	10.55
14	South Korea	Asia	77	658	2017.18	8.55
15	Turkey	Asia and Europe	62	1189	2013.89	19.18
16	Netherlands	Europe	59	705	2013.00	11.95
17	Japan	Asia	48	548	2014.17	11.42
18	Germany	Europe	47	710	2011.11	15.11
19	Saudi Arabia	Asia	45	1141	2016.96	25.36
20	Romania	Europe	35	527	2016.71	15.06
21	Egypt	Africa and Asia	33	457	2015.97	13.85
22	Belgium	Europe	31	339	2016.42	10.94
23	Pakistan	Asia	26	299	2017.38	11.50
24	Tunisia	Africa	26	343	2017.58	13.19
25	Norway	Europe	25	292	2015.80	11.68
26	Greece	Europe	24	302	2012.17	12.58
27	Finland	Europe	23	624	2013.43	27.13
28	Poland	Europe	23	392	2015.57	17.04
29	Philippines	Asia	22	629	2014.95	28.59
30	Thailand	Asia	22	173	2018.14	7.86
31	Mexico	North America	21	241	2017.43	11.48
32	Qatar	Asia	21	252	2017.62	12.00
33	South Africa	Africa	20	239	2017.10	11.95

Note: C/R = country/region, NP = number of publications, TC = total citations, APY = average publication year, CPP = citations per paper (average citations) = TC/NP.

**Table 4:** Leading institutions in PSEP based on the number of publications (minimum number of publications = 20).

Rank	Institutions	C/R	NP	TC	APY	CPP
1	China Univ. Min. & Technol.	China	73	772	2018.53	10.58
2	Mem. Univ. Newfoundland	Canada	63	1854	2014.63	29.43
3	Texas A&M Univ.	USA	44	625	2013.52	14.20
4	Univ. Loughborough	UK	44	958	2002.89	21.77
5	Delft Univ. Technol.	Netherlands	41	397	2015.17	9.68
6	Chinese Acad. Sci.	China	35	295	2017.97	8.43
7	Dalhousie Univ.	Canada	33	1229	2012.30	37.24
8	China Univ. Petr.	China	30	271	2017.27	9.03
9	Hlth & Safety Lab.	UK	30	399	2009.00	13.30
10	Univ. Leeds	UK	30	533	2004.87	17.77
11	Univ. Teknol. Malaysia	Malaysia	30	446	2015.07	14.87
12	Shandong Univ. Sci. & Technol.	China	29	730	2018.69	25.17
13	Cranfield Univ.	UK	28	512	2005.57	18.29
14	Natl. Yunlin Univ. Sci. & Technol.	Taiwan	27	249	2015.67	9.22
15	Islamic Azad Uni.v	Iran	26	544	2017.00	20.92
16	Indian Inst. Technol.	India	25	374	2013.36	14.96
17	Nanjing Tech. Univ.	China	23	74	2018.87	3.22
18	Univ. Tehran	Iran	23	509	2016.87	22.13
19	Tsinghua Univ.	China	22	327	2016.23	14.86
20	China Univ. Petr. East China	China	21	194	2017.86	9.24
21	Curtin Univ.	Australia	21	302	2016.48	14.38
22	Univ. Birmingham	UK	21	238	1997.81	11.33
23	Univ. Malaya	Malaysia	21	599	2016.38	28.52
24	Univ. Nottingham	UK	21	616	2014.00	29.33
25	Univ. Sci. & Technol. China	China	21	127	2017.71	6.05
26	Hlth & Safety Execut.	UK	20	111	2008.50	5.55
27	Univ. Sheffield	UK	20	257	2005.05	12.85

Note: C/R = country/Region, NP = number of publications, TC = total citations, APY = average publication year, CPP = citations per paper (average citations) = TC/NP.

**Table 5:** Top 25 most cited papers published in PSEP during 1990–2020 (papers were ranked with the total number of citations).

Rank	Title	Authors	Type	PY	TC	ACPY
1	A comparison of chemisorption kinetic models applied to pollutant removal on various sorbents	Ho, Y.S; McKay, G.	Article	1998	1530	66.52
2	Kinetic models for the sorption of dye from aqueous solution by wood	Ho, Y.S; McKay, G.	Article	1998	1026	44.61
3	A review on application of flocculants in wastewater treatment	Lee, C.S.; Robinson, J.; Chong, M.F.	Review	2014	362	51.71
4	A review of hazards associated with primary lithium and lithium-ion batteries	Lisbona, D.; Snee, T.	Article	2011	264	26.40
5	Treatment technologies for petroleum refinery effluents: a review	Diya’uddeen, B.H.; Daud, W.M.A.W.; Aziz, A.R.A.	Review	2011	241	24.10
6	Indicators of sustainable development for industry: a general framework	Azapagic, A.; Perdan, S.	Article	2000	240	11.43
7	Dynamic safety analysis of process systems by mapping bowtie into Bayesian network	Khakzad, N.; Khan, F.; Amyotte, P.	Article	2013	225	28.13
8	Anaerobic co-digestion of fat, oil, and grease (FOG): a review of gas production and process limitations	Long, J.H.; Aziz, T.N.; de los Reyes, F.L.; Ducoste, J.J.	Article	2012	178	19.78
9	Adsorptive removal of basic dyes from aqueous solutions by surfactant modified bentonite clay (organoclay): kinetic and competitive adsorption isotherm	Anirudhan, T.S.; Ramachandran, M.	Article	2015	168	28.00
10	Electrochemical oxidation remediation of real wastewater effluents—a review	Garcia-Segura, S.; Ocon, J.D.; Chong, M.N.	Review	2018	167	55.67
11	Catalytic pyrolysis of plastic waste: a review	Miandad, R.; Barakat, M.A.; Aburiazaiza, A.S.; Rehan, M.; Nizami, A.S.	Review	2016	162	32.40
12	Effect of pH, temperature, and air flow rate on the continuous ammonia stripping of the anaerobic digestion effluent	Gustin, S.; Marinsek-Logar, R.	Article	2011	152	15.20
13	Assessing the inherent safety of chemical process routes—is there a relation between plant costs and inherent safety	Edwards, D.W.; Lawrence, D.	Article	1993	148	5.29
14	Efficient removal of coomassie brilliant blue R-250 dye using starch/poly (alginic acid-cl-acrylamide) nanohydrogel	Sharma, G.; Naushad, M.; Kumar, A.; Rana, S.; Sharma, S.; Bhatnagar, A.; Stadler, F.J.; Ghfar, A.A.; Khan, M.R.	Article	2017	145	36.25
15	Biodiesel production from waste oil feedstocks by solid acid catalysis	Peng, B.X.; Shu, Q.; Wang, J.F.; Wang, G.R.; Wang, D.Z.; Han, M.H.	Article	2008	141	10.85
16	Systems approach to corporate sustainability—a general management framework	Azapagic, A.	Article	2003	134	7.44
17	Sustainable Industry 4.0 framework: a systematic literature review identifying the current trends and future perspectives	Kamble, S.S.; Gunasekaran, A.; Gawankar, S.A.	Review	2018	128	42.67
18	Use of membrane technology for oil field and refinery produced water treatment—a review	Munirasu, S.; Abu Haija, M.; Banat, F.	Review	2016	128	25.60
19	The diffusion behavior law of respirable dust at fully mechanized caving face in coal mine: CFD numerical simulation and engineering application	Zhou, G.; Zhang, Q.; Bai, R.N.; Fan, T.; Wang, G.;	Article	2017	121	30.25
20	Methods and models in process safety and risk management: past, present, and future	Khan, F.; Rathnayaka, S.; Ahmed, S.	Article	2015	120	20.00
21	Characterization of products from the pyrolysis of municipal solid waste	Buah, W.K.; Cunliffe, A.M.; Williams, P.T.	Article	2007	117	8.36
22	Design of water-using systems involving regeneration	Kuo, W.C.J.; Smith, R.	Article	1998	114	4.96
23	An experimental study for characterization the process of coal oxidation and spontaneous combustion by electromagnetic radiation technique	Kong, B.; Li, Z.H.; Wang, E.Y.; Lu, W.; Chen, L.; Qi, G.S.	Article	2018	109	36.33
24	Bi-level fuzzy optimization approach for water exchange in eco-industrial parks	Aviso, K.B.; Tan, R.R.; Culaba, A.B.; Cruz, J.B.	Article	2010	106	9.64
25	Harnessing methane emissions from coal mining	Warmuzinski, K.	Article	2008	106	8.15

Note: PY = publication year, TC = total citations, ACPY = average citations per year, $\mathrm{ACPY}=\frac{Total\;citations}{Current\;year-Publication\;year+1}$

**Table 6:** Highly cited references of PSEP publications ranked by the number of citations.

Rank	References	Source	Title	DT	Cluster	Citations
1	[46]	The Journal of Physical Chemistry	Over the adsorption in solution	JA	3	52
2	[74]	——	Standard methods for the examination of water and wastewater	Book	5	48
3	[47]	Royal Swedish Academy of Sciences	About the theory of so-called adsorption of soluble substances	JA	3	48
4	[49]	Journal of the American Chemical society	The adsorption of gases on plane surfaces of glass, mica, and platinum	JA	3	45
5	[69]	——	Loss prevention in the process industry	Book	2	45
6	[50]	Process biochemistry	Pseudo-second order model for sorption processes	JA	3	41
7	[48]	Journal of the sanitary engineering division	Kinetics of adsorption on carbon from solution	JA	3	32
8	[55]	Process Safety and Environmental Protection	Dynamic safety analysis of process systems by mapping bowtie into Bayesian network	JA	1	27
9	[53]	Journal of the American chemical society	The constitution and fundamental properties of solids and liquids. Part I. Solids	JA	3	26
10	[71]	——	Guidelines for chemical process quantitative risk analysis	Book	2	25
11	[59]	Information and Control	Fuzzy sets	JA	1	25
12	[79]	Fuel	An intelligent gel designed to control the spontaneous combustion of coal: fire prevention and extinguishing properties	JA	6	24
13	[58]	Process safety and environmental protection	Methods and models in process safety and risk management: past, present, and future	JA	1	24
14	[75]	——	Standard methods for the examination of water and wastewater	Book	5	23
15	[72]	——	Dust explosions in the process industries	Book	2	23
16	[63]	Process Safety and Environmental Protection	Assessing the inherent safety of chemical process routes: is there a relation between plant costs and inherent safety?	JA	4	22
17	[82]	——	Human error	Book	1	21
18	[76]	——	Standard methods for the examination of water and wastewater	Book	5	20
19	[61]	Reliability Engineering & System Safety	Safety analysis in process facilities: comparison of fault tree and Bayesian network approaches	JA	1	20
20	[67]	Industrial & Engineering Chemistry Research	Assessing safety, health, and environmental impact early during process development	JA	4	18
21	[52]	Journal of environmental management	Removal of heavy metal ions from wastewaters: a review	JA	3	18
22	[70]	——	Chemical process safety: fundamentals with applications	Book	2	17
23	[64]	Process Safety Progress	Multivariate hazard identification and ranking system	JA	4	17
24	[68]	Process Safety and Environmental Protection	Safety weighted hazard index (SWeHI): a new, user-friendly tool for swift yet comprehensive hazard identification and safety evaluation in chemical process industries	JA	4	17
25	[83]	Safety science	Risk management in a dynamic society: a modelling problem	JA	1	17
26	[60]	Process safety and environmental protection	SHIPP methodology: Predictive accident modeling approach. Part I: Methodology and model description	JA	1	17
27	[78]	Journal of hazardous materials	Landfill leachate treatment: review and opportunity	JA	5	17
28	[84]	Chemical Engineering Science	Wastewater minimization	JA	1	17
29	[62]	Reliability Engineering & System Safety	Improving the analysis of dependable systems by mapping fault trees into Bayesian networks	JA	1	16
30	[77]	Water research	Recent developments in photocatalytic water treatment technology: a review	JA	5	16
31	[85]	Analytical chemistry	Colorimetric method for determination of sugars and related substances	JA	5	16
32	[80]	Advanced Powder Technology	Effects of air volume ratio parameters on air curtain dust suppression in a rock tunnel’s fully mechanized working face	JA	6	16
33	[86]	Journal of hazardous materials	Escalation thresholds in the assessment of domino accidental events	JA	2	15
34	[87]	Journal of hazardous materials	Domino effect in chemical accidents: main features and accident sequences	JA	2	15
35	[51]	Chemical engineering journal	Insights into the modeling of adsorption isotherm systems	JA	3	15
36	[65]	Journal of Hazardous Materials	A simple graphical method for measuring inherent safety	JA	4	15
37	[81]	International journal of coal geology	Coal mine methane: a review of capture and utilization practices with benefits to mining safety and to greenhouse gas reduction	JA	6	15
38	[56]	Reliability Engineering & System Safety	Dynamic risk analysis using bowtie approach	JA	1	15
39	[57]	Journal of loss Prevention in the Process Industries	Techniques and methodologies for risk analysis in chemical process industries	JA	1	15
40	[73]	Journal of Loss Prevention in the process Industries	Major accidents in process industries and an analysis of causes and consequences	JA	2	15
41	[66]	Journal of Loss Prevention in the Process Industries	I2SI: a comprehensive quantitative tool for inherent safety and cost evaluation	JA	4	15
42	[54]	Chemical engineering science	Plant-specific dynamic failure assessment using Bayesian theory	JA	1	15
43	[88]	Industrial & Engineering Chemistry Fundamentals	A new two-constant equation of state	JA	2	15

Note: Cluster matches the color for each cluster group in Figure 7, DT = document type, JA = journal articles.

**Table 7:** Top 10 keywords for PSEP in terms of various periods.

R	Before 2008		2008–2012		2013–2017		2018–2020	
	Keyword	F	Keyword	F	Keyword	F	Keyword	F
1	Risk	40	Risk assessment	73	Adsorption	122	Microbial community	14
2	LCA	24	Safety	56	Kinetics	80	CCD	13
3	Combustion	17	Modeling	46	RSM	74	CSC	12
4	HAZOP	15	Explosion	43	Optimization	67	Ionic liquid	12
5	Runaway reaction	15	Inherent safety	34	Heavy metals	56	Toxicity	11
6	Health	14	Anaerobic digestion	31	CFD	49	Forward osmosis	11
7	Sustainable development	12	Recycling	27	Wastewater treatment	44	Sensitivity analysis	9
8	Radiation	11	Biosorption	23	Photocatalysis	43	Industry 4.0	8
9	Incineration	11	Consequence analysis	23	Wastewater	43	Air leakage	7
10	Pollution	9	Human factors	22	Process safety	43	Microwave	7
11	Environmental impact	9	Regeneration	21	Activated carbon	40	Spontaneous coal combustion	7
12	Offshore	8	Biomass	21	Numerical simulation	37	Process optimization	7
13	Effluent	7	Sustainability	21	Bayesian network	34	Coal mine	7
14	Transportation	7	Activated sludge	20	Biodiesel	34	Catalytic ozonation	7
15	Atrazine	7	Environment	20	Isotherm	34	Coal and gas outburst	6
16	LPG	7	Gas explosion	19	Dust explosion	34	Synergistic effect	6
17	Phosphorus removal	7	J-value	19	AOP	32	Drinking water	6
18	Major hazards	7	Mathematical modeling	18	Electrocoagulation	31	Moisture content	6
19	Decision making	7	Process design	17	Biodegradation	30	Mineralization	6
20	Waste incineration	7	Accident	17	Pyrolysis	28	Electro-oxidation	5

Note: R = rank; F = frequency of each keyword; LCA = lifecycle assessment; HAZOP = hazard and operability studies; LPG = liquefied petroleum gas; RSM = response surface methodology; CFD = computational fluid dynamics; AOP = advanced oxidation process; CCD = central composite design; CSC = coal spontaneous combustion.

## References

[1] Merigó J.M., Miranda J., Modak N.M., Boustras G., de la Sotta C. (2019). Forty years of Safety Science: A bibliometric overview. Saf. Sci..

[2] Singh S., Dhir S., Das V.M., Sharma A. (2020). Bibliometric overview of the Technological Forecasting and Social Change journal: Analysis from 1970 to 2018. Technol. Forecast. Soc. Change.

[3] Broadus R.N. (1987). Toward a definition of “bibliometrics”. Scientometrics.

[4] Wang C., Lim M.K., Zhao L., Tseng M.-L., Chien C.-F., Lev B. (2020). The evolution of omega-the international journal of management science over the past 40 years: A bibliometric overview. Omega.

[5] Aria M., Cuccurullo C. (2017). bibliometrix: An R-tool for comprehensive science mapping analysis. J. Informetr..

[6] Bindra S., Parameswar N., Dhir S. (2019). Strategic management: The evolution of the field. Strategic Change.

[7] Singh S., Akbani I., Dhir S. (2020). Service innovation implementation: A systematic review and research agenda. Serv. Ind. J..

[8] Li J., Wang Y., Yan B. (2018). The hotspots of life cycle assessment for bioenergy: A review by social network analysis. Sci. Total Environ..

[9] Zhi W., Ji G. (2012). Constructed wetlands, 1991–2011: A review of research development, current trends, and future directions. Sci. Total Environ..

[10] Mao G., Hu H., Liu X., Crittenden J., Huang N. (2021). A bibliometric analysis of industrial wastewater treatments from 1998 to 2019. Environ. Pollut..

[11] Li J., Goerlandt F., Reniers G. (2020). An overview of scientometric mapping for the Safety Science community: Methods, tools, and processes. Saf. Sci..

[12] Krauskopf E. (2018). A bibiliometric analysis of the Journal of Infection and Public Health: 2008–2016. J. Infect. Public Health.

[13] Laengle S., Modak N.M., Merigo J.M., Zurita G. (2018). Twenty-five years of group decision and negotiation: A bibliometric overview. Gr. Decis. Negot..

[14] Flores P. (2019). The journal of Mechanism and Machine Theory: Celebrating 55 years since its foundation. Mech. Mach. Theory.

[15] Laengle S., Merigó J.M., Miranda J., Słowiński R., Bomze I., Borgonovo E., Dyson R.G., Oliveira J.F., Teunter R. (2017). Forty years of the European Journal of Operational Research: A bibliometric overview. Eur. J. Oper. Res..

[16] Ji L., Liu C., Huang L., Huang G. (2018). The evolution of resources conservation and recycling over the past 30 years: A bibliometric overview. Resour. Conserv. Recycl..

[17] Modak N.M., Merigó J.M., Weber R., Manzor F., Ortúzar J.d.D. (2019). Fifty years of Transportation Research journals: A bibliometric overview. Transp. Res. Part A Policy Pract..

[18] Cancino C., Merigó J.M., Coronado F., Dessouky Y., Dessouky M. (2017). Forty years of computers & industrial engineering: A bibliometric analysis. Comput. Ind. Eng..

[19] Cobo M.J., Martínez M.A., Gutiérrez-Salcedo M., Fujita H., Herrera-Viedma E. (2015). 25 years at knowledge-based systems: A bibliometric analysis. Knowl. Based Syst..

[20] Tang M., Liao H., Su S.-F. (2018). A Bibliometric overview and visualization of the international journal of fuzzy systems between 2007 and 2017. Int. J. Fuzzy Syst..

[21] Wang C., Zhao L., Vilela A.L.M., Lim M.K. (2019). The evolution of Industrial Management & Data Systems over the past 25 years. Ind. Manag. Data Syst..

[22] Van Eck N.J., Waltman L. (2010). Software survey: VOSviewer, a computer program for bibliometric mapping. Scientometrics.

[23] Mingers J., Leydesdorff L. (2015). A review of theory and practice in scientometrics. Eur. J. Oper. Res..

[24] Merigó J.M., Herrera-Viedma E., Cobo M.J., Laengle S., Rivas D. (2017). A Bibliometric analysis of the first twenty years of soft computing. Advances in Fuzzy Logic and Technology 2017.

[25] Van Nunen K., Li J., Reniers G., Ponnet K. (2018). Bibliometric analysis of safety culture research. Saf. Sci..

[26] Yang Y., Chen G., Reniers G., Goerlandt F. (2020). A bibliometric analysis of process safety research in China:Understanding safety research progress as a basis for making China’s chemical industry more sustainable. J. Cleaner Prod..

[27] Li J. (2017). Scientometrics and Knowledge Network Analysis.

[28] Li J. (2018). Principles and Applications of Mapping Knowledge Domains: A Beginner’s Guide to VOSviewer and CitNetExplorer.

[29] Verrall B., Pickering C.M. (2020). Alpine vegetation in the context of climate change: A global review of past research and future directions. Sci. Total Environ..

[30] Nazaripour M., Reshadi M.A.M., Mirbagheri S.A., Nazaripour M., Bazargan A. (2021). Research trends of heavy metal removal from aqueous environments. J. Environ. Manag..

[31] Su Y., Yu Y., Zhang N. (2020). Carbon emissions and environmental management based on Big Data and Streaming Data: A bibliometric analysis. Sci. Total Environ..

[32] Padilla F.M., Gallardo M., Manzano-Agugliaro F. (2018). Global trends in nitrate leaching research in the 1960–2017 period. Sci. Total Environ..

[33] Li H., Jiang H.D., Yang B., Liao H. (2019). An analysis of research hotspots and modeling techniques on carbon capture and storage. Sci. Total Environ..

[34] Vanzetto G.V., Thome A. (2019). Bibliometric study of the toxicology of nanoescale zero valent iron used in soil remediation. Environ. Pollut..

[35] Jin R., Zou P.X.W., Piroozfar P., Wood H., Yang Y., Yan L., Han Y. (2019). A science mapping approach based review of construction safety research. Saf. Sci..

[36] Akram R., Thaheem M.J., Nasir A.R., Ali T.H., Khan S. (2019). Exploring the role of building information modeling in construction safety through science mapping. Saf. Sci..

[37] Amin M.T., Khan F., Amyotte P. (2019). A bibliometric review of process safety and risk analysis. Process Saf. Environ. Protect..

[38] Li J., Reniers G., Cozzani V., Khan F. (2017). A bibliometric analysis of peer-reviewed publications on domino effects in the process industry. J. Loss Prev. Process Ind..

[39] Yang Y., Reniers G., Chen G., Goerlandt F. (2019). A bibliometric review of laboratory safety in universities. Saf. Sci..

[40] Zou X., Yue W.L., Vu H.L. (2018). Visualization and analysis of mapping knowledge domain of road safety studies. Accid. Anal. Prev..

[41] Liu H., Chen H., Hong R., Liu H., You W. (2020). Mapping knowledge structure and research trends of emergency evacuation studies. Saf. Sci..

[42] Tao J., Yang F., Qiu D., Reniers G. (2020). Analysis of safety leadership using a science mapping approach. Process Saf. Environ. Protect..

[43] Merigó J.M., Gil-Lafuente A.M., Yager R.R. (2015). An overview of fuzzy research with bibliometric indicators. Appl. Soft Comput..

[44] Merigó J.M., Mas-Tur A., Roig-Tierno N., Ribeiro-Soriano D. (2015). A bibliometric overview of the Journal of Business Research between 1973 and 2014. J. Bus. Res..

[45] Ho Y., McKay G. (1998). A comparison of chemisorption kinetic models applied to pollutant removal on various sorbents. Process Saf. Environ. Protect..

[46] Freundlich H. (1906). Over the adsorption in solution. J. Phys. Chem..

[47] Lagergren S. (1898). About the theory of so called adsorption of soluble substances. Kungliga Sven. Vetensk. Handl..

[48] Weber W.J., Morris J.C. (1963). Kinetics of adsorption on carbon from solution. J. Sanit. Eng. Div..

[49] Langmuir I. (1918). The adsorption of gases on plane surfaces of glass, mica and platinum. J. Am. Chem. Soc..

[50] Ho Y.-S., McKay G. (1999). Pseudo-second order model for sorption processes. Process Biochem..

[51] Foo K.Y., Hameed B.H. (2010). Insights into the modeling of adsorption isotherm systems. Chem. Eng. J..

[52] Fu F., Wang Q. (2011). Removal of heavy metal ions from wastewaters: A review. J. Environ. Manag..

[53] Langmuir I. (1916). The constitution and fundamental properties of solids and liquids. Part, I. Solids. J. Am. Chem. Soc..

[54] Meel A., Seider W.D. (2006). Plant-specific dynamic failure assessment using Bayesian theory. Chem. Eng. Sci..

[55] Khakzad N., Khan F., Amyotte P. (2013). Dynamic safety analysis of process systems by mapping bow-tie into Bayesian network. Process Saf. Environ. Protect..

[56] Khakzad N., Khan F., Amyotte P. (2012). Dynamic risk analysis using bow-tie approach. Reliab. Eng. Syst. Saf..

[57] Khan F.I., Abbasi S. (1998). Techniques and methodologies for risk analysis in chemical process industries. J. Loss Prev. Process Ind..

[58] Khan F., Rathnayaka S., Ahmed S. (2015). Methods and models in process safety and risk management: Past, present and future. Process Saf. Environ. Prot..

[59] Zadeh L.A. (1965). Fuzzy sets. Inf. Control.

[60] Rathnayaka S., Khan F., Amyotte P. (2011). SHIPP methodology: Predictive accident modeling approach. Part I: Methodology and model description. Process Saf. Environ. Prot..

[61] Khakzad N., Khan F., Amyotte P. (2011). Safety analysis in process facilities: Comparison of fault tree and Bayesian network approaches. Reliab. Eng. Syst. Saf..

[62] Bobbio A., Portinale L., Minichino M., Ciancamerla E. (2001). Improving the analysis of dependable systems by mapping fault trees into Bayesian networks. Reliab. Eng. Syst. Saf..

[63] Edwards D.W., Lawrence D. (1993). Assessing the inherent safety of chemical process routes: Is there a relation between plant costs and inherent safety?. Process Saf. Environ. Prot..

[64] Khan F.I., Abbasi S. (1998). Multivariate hazard identification and ranking system. Process Saf. Prog..

[65] Gupta J., Edwards D.W. (2003). A simple graphical method for measuring inherent safety. J. Hazard. Mater..

[66] Khan F.I., Amyotte P.R. (2005). I2SI: A comprehensive quantitative tool for inherent safety and cost evaluation. J. Loss Prev. Process Ind..

[67] Koller G., Fischer U., Hungerbühler K. (2000). Assessing safety, health, and environmental impact early during process development. Ind. Eng. Chem. Res..

[68] Khan F.I., Husain T., Abbasi S.A. (2001). Safety weighted hazard index (SWeHI): A new, user-friendly tool for swift yet comprehensive hazard identification and safety evaluation in chemical process industries. Process Saf. Environ. Prot..

[69] Lees F.P. (1996). Loss Prevention in the Process Industries.

[70] Crowl D.A., Louvar J.F. (2011). Chemical Process Safety Fundamentals with Applications.

[71] CCPS (2000). Guidelines for Chemical Process Quantitative Risk Analysis.

[72] Eckhoff R.K. (2003). Dust explosions in the Process Industries.

[73] Khan F.I., Abbasi S. (1999). Major accidents in process industries and an analysis of causes and consequences. J. Loss Prev. Process Ind..

[74] APHA (2005). Standard Methods for the Examination of Water and Wastewater.

[75] APHA (1998). Standard Methods for the Examination of Water and Wastewater.

[76] APHA (2012). Standard Methods for the Examination of Water and Wastewater.

[77] Chong M.N., Jin B., Chow C.W., Saint C. (2010). Recent developments in photocatalytic water treatment technology: A review. Water Res..

[78] Renou S., Givaudan J., Poulain S., Dirassouyan F., Moulin P. (2008). Landfill leachate treatment: Review and opportunity. J. Hazard. Mater..

[79] Cheng W., Hu X., Xie J., Zhao Y. (2017). An intelligent gel designed to control the spontaneous combustion of coal: Fire prevention and extinguishing properties. Fuel.

[80] Wang H., Nie W., Cheng W., Liu Q., Jin H. (2018). Effects of air volume ratio parameters on air curtain dust suppression in a rock tunnel’s fully-mechanized working face. Adv. Powder Technol..

[81] Karacan C.Ö., Ruiz F.A., Cotè M., Phipps S. (2011). Coal mine methane: A review of capture and utilization practices with benefits to mining safety and to greenhouse gas reduction. Int. J. Coal Geol..

[82] Reason J. (1990). Human Error.

[83] Rasmussen J. (1997). Risk management in a dynamic society: A modelling problem. Safety Sci..

[84] Wang Y.P., Smith R. (1994). Wastewater Minimization. Chem. Eng. Sci..

[85] Dubois M., Gilles K.A., Hamilton J.K., Rebers P.t., Smith F. (1956). Colorimetric method for determination of sugars and related substances. Anal. Chem..

[86] Cozzani V., Gubinelli G., Salzano E. (2006). Escalation thresholds in the assessment of domino accidental events. J. Hazard. Mater..

[87] Darbra R., Palacios A., Casal J. (2010). Domino effect in chemical accidents: Main features and accident sequences. J. Hazard. Mater..

[88] Peng D.-Y., Robinson D.B. (1976). A new two-constant equation of state. Ind. Eng. Chem. Fundam..

[89] Nasserzadeh V., Swithenbank J., Lawrence D., Garrod N.P. (1995). Emission testing and design optimization of the Sheffield clinical incinerator plant. Process Saf. Environ. Prot..

[90] Jørgensen K., Madsen O.H. (2000). Modern control systems for MSW plants. Process Saf. Environ. Prot..

[91] Nasserzadeh V., Swithenbank J., Schofield C., Scott D.W., Loader A., Leonard A., Russell R., Winn D. (1993). Three-dimensional modelling of the Coventry MSW incinerator using computational fluid dynamics and experimental data. Process Saf. Environ. Prot. Trans. Inst. Chem. Eng. Part B.

[92] Goh Y.R., Lim C.N., Zakaria R., Chan K.H., Reynolds G., Yang Y.B., Siddall R.G., Nasserzadeh V., Swithenbank J. (2000). Mixing, Modelling and measurements of incinerator bed combustion. Process Saf. Environ. Prot..

[93] Zheng G., Di Lalla S., Koziński J. (1998). Experimental methodology and determination of optimum operating parameters during solid waste burning. Process Saf. Environ. Prot..

[94] Choy K.K.H., Ko D.C.K., Cheung W.-H., Fung J.S.C., Hui D.C.W., Porter J.F., Mckay G. (2004). Municipal solid waste utilization for integrated cement processing with waste minimization: A pilot scale proposal. Process Saf. Environ. Prot..

[95] Wayman M., Chen S., Doan K. (1993). Production of fuel ethanol from the waste paper in garbage. Process Saf. Environ. Prot..

[96] Ward D., Goh Y., Clarkson P., Lee P., Nasserzadeh V., Swithenbank J. (2002). A novel energy-efficient process utilizing regenerative burners for the detoxification of fly ash. Process Saf. Environ. Prot..

[97] Chagger H., Jones J., Pourkashanian M., Williams A. (2000). The formation of VOC, PAH and dioxins during incineration. Process Saf. Environ. Prot..

[98] Lee P., Nasserzadeh V., Swithenbank J., Laming J., Goodfellow J., Mcleod C., Argent B., Lawrence D., Garrod N. (1999). Sintering of the APC residue from municipal waste incinerators. Process Saf. Environ. Prot..

[99] Golonka K.A., Brennan D.J. (1997). Costs and environmental impacts in pollutant treatment: A case study of sulphur dioxide emissions from metallurgical smelters. Process Saf. Environ. Prot..

[100] Narayanan D., Zhang Y., Mannan M.S. (2007). Engineering for Sustainable Development (ESD) in bio-diesel production. Process Saf. Environ. Prot..

[101] Azapagic A. (2003). Systems approach to corporate sustainability: A general management framework. Process Saf. Environ. Prot..

[102] Azapagic A., Perdan S. (2000). Indicators of sustainable development for industry: A general framework. Process Saf. Environ. Prot..

[103] Cullis C.F., Hirschler M.M., Stroud M.A.M. (1995). Control of solid and gaseous pollutants formed during diesel fuel combustion. Process Saf. Environ. Prot..

[104] Andrews J., Smith R., Gregory J. (1994). Procedure to calculate the explosion frequency for a module on an offshore platform. Process Saf. Environ. Prot. Trans. Inst. Chem. Eng. Part B.

[105] Pula R., Khan F.I., Veitch B., Amyotte P.R. (2006). A Grid based approach for fire and explosion consequence analysis. Process Saf. Environ. Prot..

[106] Haastrup P., Rasmussen K. (1994). A study of f-N curves for accidents involving highly flammable gases and some toxic gases. Process Saf. Environ. Prot..

[107] Roberts T., Gosse A., Hawksworth S. (2000). Thermal radiation from fireballs on failure of liquefied petroleum gas storage vessels. Process Saf. Environ. Prot..

[108] Khan F.I., Sadiq R., Haddara M.M. (2004). Risk-Based Inspection and Maintenance (RBIM): Multi-attribute decision-making with aggregative risk analysis. Process Saf. Environ. Prot..

[109] Ferdous R., Khan F.I., Veitch B., Amyotte P.R. (2007). Methodology for computer-aided fault tree analysis. Process Saf. Environ. Prot..

[110] Woods H.F. (1995). Assessing the risks to human health associated with exposure to dioxins. Process Saf. Environ. Prot..

[111] Rew P.J., Hulbert W.G., Deaves D.M. (1997). Modelling of thermal radiation from external hydrocarbon pool fires. Process Saf. Environ. Prot..

[112] Snee T.J., Cusco L. (2005). Pilot-Scale Evaluation of the Inhibition of Exothermic Runaway. Process Saf. Environ. Prot..

[113] Sarkar C., Abbasi S.A. (2006). Enhancing the accuracy of forecasting impact of accidents in chemical process industry by the application of cellular automata technique. Process Saf. Environ. Prot..

[114] Ménard Y., Asthana A., Patisson F., Sessiecq P., Ablitzer D. (2006). Thermodynamic study of heavy metals behaviour during municipal waste incineration. Process Saf. Environ. Prot..

[115] Doyle J.D., Philp R., Churchley J., Parsons S.A. (2000). Analysis of struvite precipitation in real and synthetic liquors. Process Saf. Environ. Prot..

[116] Kolaczkowski S.T., Perera S.P., Crittenden B.D., Rankin A.J., Hayes R.E. (1994). Catalytic combustion of polychlorinated biphenyls in monolith reactors. Process Saf. Environ. Prot..

[117] Peregrina C.A., Lecomte D., Arlabosse P., Rudolph V. (2006). Life Cycle Assessment (LCA) applied to the design of an innovative drying process for sewage sludge. Process Saf. Environ. Prot..

[118] Nicholas M.J., Clift R., Azapagic A., Walker F.C., Porter D.E. (2000). Determination of ‘Best Available Techniques’ for integrated pollution prevention and control: A life cycle approach. Process Saf. Environ. Prot..

[119] McCoy S.A., Wakeman S.J., Larkin F.D., Chung P.W.H., Rushton A.G., Lees F.P. (2000). Hazid, A computer aid for hazard identification: 4. Learning set, main study system, output quality and validation trials. Process Saf. Environ. Prot..

[120] Švandová Z., Jelemenský L., Markoš J., Molnár A. (2005). Steady states analysis and dynamic simulation as a complement in the hazop study of chemical reactors. Process Saf. Environ. Prot..

[121] Harris J. (2002). On system condition auditing. Process Saf. Environ. Prot..

[122] Paralikas A.N., Lygeros A.I. (2005). A multi-criteria and fuzzy logic based methodology for the relative ranking of the fire hazard of chemical substances and installations. Process Saf. Environ. Prot..

[123] Hall G.M., Howe J. (2012). Energy from waste and the food processing industry. Process Saf. Environ. Prot..

[124] Long J.H., Aziz T.N., Francis III L., Ducoste J.J. (2012). Anaerobic co-digestion of fat, oil, and grease (FOG): A review of gas production and process limitations. Process Saf. Environ. Prot..

[125] Kralj D. (2009). Experimental study of recycling lightweight concrete with aggregates containing expanded glass. Process Saf. Environ. Prot..

[126] Aviso K.B., Tan R.R., Culaba A.B., Cruz J.B. (2010). Bi-level fuzzy optimization approach for water exchange in eco-industrial parks. Process Saf. Environ. Prot..

[127] Lin Y.-H., Zheng H.-X., Juan M.-L. (2012). Biohydrogen production using waste activated sludge as a substrate from fructose-processing wastewater treatment. Process Saf. Environ. Prot..

[128] Huang M.-h., Yang Y.-d., Chen D.-h., Chen L., Guo H.-d. (2012). Removal mechanism of trace oxytetracycline by aerobic sludge. Process Saf. Environ. Prot..

[129] Chen K., Lei H., Li Y., Li H., Zhang X., Yao C. (2011). Physical and chemical characteristics of waste activated sludge treated with electric field. Process Saf. Environ. Prot..

[130] Papalexandrou M.A., Pilavachi P.A., Chatzimouratidis A.I. (2008). Evaluation of liquid bio-fuels using the Analytic Hierarchy Process. Process Saf. Environ. Prot..

[131] Kossoy A.A., Akhmetshin Y.G. (2012). Simulation-based approach to design of inherently safer processes. Process Saf. Environ. Prot..

[132] Banimostafa A., Papadokonstantakis S., Hungerbühler K. (2012). Evaluation of EHS hazard and sustainability metrics during early process design stages using principal component analysis. Process Saf. Environ. Prot..

[133] Hassim M.H., Hurme M. (2010). Occupational chemical exposure and risk estimation in process development and design. Process Saf. Environ. Prot..

[134] Atkinson G., Cusco L. (2011). Buncefield: A violent, episodic vapour cloud explosion. Process Saf. Environ. Prot..

[135] Cheng J.-w., Yang S.-q. (2011). Improved Coward explosive triangle for determining explosibility of mixture gas. Process Saf. Environ. Prot..

[136] Hu J., Zhang L., Liang W. (2012). Opportunistic predictive maintenance for complex multi-component systems based on DBN-HAZOP model. Process Saf. Environ. Prot..

[137] Falcke T.J., Hoadley A.F.A., Brennan D.J., Sinclair S.E. (2011). The sustainability of clean coal technology: IGCC with/without CCS. Process Saf. Environ. Prot..

[138] Vaughen B.K., Klein J.A. (2012). What you don’t manage will leak: A tribute to Trevor Kletz. Process Saf. Environ. Prot..

[139] Thomas P.J., Taylor R.H. (2012). J-value analysis of different regulatory limits for workers and the public. Process Saf. Environ. Prot..

[140] Thomas P.J., Jones R.D. (2010). Extending the J-value framework for safety analysis to include the environmental costs of a large accident. Process Saf. Environ. Prot..

[141] Guštin S., Marinšek-Logar R. (2011). Effect of pH, temperature and air flow rate on the continuous ammonia stripping of the anaerobic digestion effluent. Process Saf. Environ. Prot..

[142] Doan H.D., Lohi A., Dang V.B.H., Dang-Vu T. (2008). Removal of Zn+2 and Ni+2 by adsorption in a fixed bed of wheat straw. Process Saf. Environ. Prot..

[143] Dellavedova M., Derudi M., Biesuz R., Lunghi A., Rota R. (2012). On the gasification of biomass: Data analysis and regressions. Process Saf. Environ. Prot..

[144] An H.-S., Park S.-S., Kim K.-H., Park S.-U., Ohm T.-I. (2011). Treatment of PCB-contaminated pole transformers by vacuum thermal recycling with voltage adjuster. Process Saf. Environ. Prot..

[145] Coldrick S., Gant S.E., Atkinson G.T., Dakin R. (2011). Factors affecting vapour production in large scale evaporating liquid cascades. Process Saf. Environ. Prot..

[146] Arslan O. (2009). Quantitative evaluation of precautions on chemical tanker operations. Process Saf. Environ. Prot..

[147] Ferdous R., Khan F., Sadiq R., Amyotte P., Veitch B. (2009). Handling data uncertainties in event tree analysis. Process Saf. Environ. Prot..

[148] Azapagic A., Chalabi Z., Fletcher T., Grundy C., Jones M., Leonardi G., Osammor O., Sharifi V., Swithenbank J., Tiwary A. (2013). An integrated approach to assessing the environmental and health impacts of pollution in the urban environment: Methodology and a case study. Process Saf. Environ. Prot..

[149] Arenas C.N., Vasco A., Betancur M., Martínez J.D. (2017). Removal of indigo carmine (IC) from aqueous solution by adsorption through abrasive spherical materials made of rice husk ash (RHA). Process Saf. Environ. Prot..

[150] Zanin E., Scapinello J., de Oliveira M., Rambo C.L., Franscescon F., Freitas L., de Mello J.M.M., Fiori M.A., Oliveira J.V., Dal Magro J. (2017). Adsorption of heavy metals from wastewater graphic industry using clinoptilolite zeolite as adsorbent. Process Saf. Environ. Prot..

[151] Demirbas E., Kobya M. (2017). Operating cost and treatment of metalworking fluid wastewater by chemical coagulation and electrocoagulation processes. Process Saf. Environ. Prot..

[152] Daud N.M., Sheikh Abdullah S.R., Abu Hasan H., Yaakob Z. (2015). Production of biodiesel and its wastewater treatment technologies: A review. Process Saf. Environ. Prot..

[153] Aziz H.A., Shariff A.M., Rusli R., Yew K.H. (2014). Managing process chemicals, technology and equipment information for pilot plant based on Process Safety Management standard. Process Saf. Environ. Prot..

[154] Yuan Z., Khakzad N., Khan F., Amyotte P. (2016). Domino effect analysis of dust explosions using Bayesian networks. Process Saf. Environ. Prot..

[155] Bonvicini S., Antonioni G., Morra P., Cozzani V. (2015). Quantitative assessment of environmental risk due to accidental spills from onshore pipelines. Process Saf. Environ. Prot..

[156] Bubbico R., Carbone F., Ramírez-Camacho J.G., Pastor E., Casal J. (2016). Conditional probabilities of post-release events for hazardous materials pipelines. Process Saf. Environ. Prot..

[157] Abuswer M., Amyotte P., Khan F., Imtiaz S. (2016). Retrospective risk analysis and controls for Semabla grain storage hybrid mixture explosion. Process Saf. Environ. Prot..

[158] Yang Q., Zhong Y., Zhong H., Li X., Du W., Li X., Chen R., Zeng G. (2015). A novel pretreatment process of mature landfill leachate with ultrasonic activated persulfate: Optimization using integrated Taguchi method and response surface methodology. Process Saf. Environ. Prot..

[159] Aziz A.R.A., Asaithambi P., Daud W.M.A.B.W. (2016). Combination of electrocoagulation with advanced oxidation processes for the treatment of distillery industrial effluent. Process Saf. Environ. Prot..

[160] Sivakumar Natarajan T., Bajaj H.C., Tayade R.J. (2016). Palmyra tuber peel derived activated carbon and anatase TiO_2_ nanotube based nanocomposites with enhanced photocatalytic performance in rhodamine 6G dye degradation. Process Saf. Environ. Prot..

[161] Matsushita T., Hirai S., Ishikawa T., Matsui Y., Shirasaki N. (2015). Decomposition of 1,4-dioxane by vacuum ultraviolet irradiation: Study of economic feasibility and by-product formation. Process Saf. Environ. Prot..

[162] Gajera H.P., Bambharolia R.P., Hirpara D.G., Patel S.V., Golakiya B.A. (2015). Molecular identification and characterization of novel Hypocrea koningii associated with azo dyes decolorization and biodegradation of textile dye effluents. Process Saf. Environ. Prot..

[163] Wang Y., Sun S., Yang F., Li S., Wu J., Liu J., Zhong S., Zeng J. (2015). The effects of activated Al_2_O_3_ on the recycling of light oil from the catalytic pyrolysis of waste printed circuit boards. Process Saf. Environ. Prot..

[164] Jardak K., Dirany A., Drogui P., El Khakani M.A. (2017). Statistical optimization of electrochemical oxidation of ethylene glycol using response surface methodology. Process Saf. Environ. Prot..

[165] Bernechea E.J., Arnaldos J. (2014). Optimizing the design of storage facilities through the application of ISD and QRA. Process Saf. Environ. Prot..

[166] González Dan J.R., Guix A., Martí V., Arnaldos J., Darbra R.M. (2016). Monte Carlo simulation as a tool to show the influence of the human factor into the quantitative risk assessment. Process Saf. Environ. Prot..

[167] Eini S., Shahhosseini H.R., Javidi M., Sharifzadeh M., Rashtchian D. (2016). Inherently safe and economically optimal design using multi-objective optimization: The case of a refrigeration cycle. Process Saf. Environ. Prot..

[168] Lavasani S.M., Zendegani A., Celik M. (2015). An extension to Fuzzy Fault Tree Analysis (FFTA) application in petrochemical process industry. Process Saf. Environ. Prot..

[169] Widiatmojo A., Sasaki K., Sugai Y., Suzuki Y., Tanaka H., Uchida K., Matsumoto H. (2015). Assessment of air dispersion characteristic in underground mine ventilation: Field measurement and numerical evaluation. Process Saf. Environ. Prot..

[170] Haoran Z., Sanmiquel Pera L., Zhao Y., Vintro Sanchez C. (2015). Researches and applications on geostatistical simulation and laboratory modeling of mine ventilation network and gas drainage zone. Process Saf. Environ. Prot..

[171] Abu Bakar S.N.H., Abu Hasan H., Mohammad A.W., Abdullah S.R.S., Ngteni R., Yusof K.M.M. (2020). Performance of a laboratory-scale moving bed biofilm reactor (MBBR) and its microbial diversity in palm oil mill effluent (POME) treatment. Process Saf. Environ. Prot..

[172] Botta L.S., Delforno T.P., Rabelo C.A.B.S., Silva E.L., Varesche M.B.A. (2020). Microbial community analyses by high-throughput sequencing of rumen microorganisms fermenting office paper in mesophilic and thermophilic lysimeters. Process Saf. Environ. Prot..

[173] Xi X., Shi Q., Jiang S., Zhang W., Wang K., Zhengyan W. (2020). Study on the effect of ionic liquids on coal spontaneous combustion characteristic by microstructure and thermodynamic. Process Saf. Environ. Prot..

[174] Andrade L.R.S., Cruz I.A., de Melo L., Vilar D.d.S., Fuess L.T., Reis e Silva G., Silva Manhães V.M., Torres N.H., Soriano R.N., Bharagava R.N. (2020). Oyster shell-based alkalinization and photocatalytic removal of cyanide as low-cost stabilization approaches for enhanced biogas production from cassava starch wastewater. Process Saf. Environ. Prot..

[175] Thanekar P., Lakshmi N., Shah M., Gogate P.R., Znak Z., Sukhatskiy Y., Mnykh R. (2020). Degradation of dimethoate using combined approaches based on hydrodynamic cavitation and advanced oxidation processes. Process Saf. Environ. Prot..

[176] Shukla N., Dhawan N. (2020). Rapid microwave processing of discarded tubular lights for extraction of rare earth values. Process Saf. Environ. Prot..

[177] Cheng G., Li Z., Sun L., Li Y., Fu J. (2020). Application of microwave/electrodeless discharge ultraviolet/ozone sterilization technology in water reclamation. Process Saf. Environ. Prot..

[178] Shahi N.K., Maeng M., Kim D., Dockko S. (2020). Removal behavior of microplastics using alum coagulant and its enhancement using polyamine-coated sand. Process Saf. Environ. Prot..

[179] Zhao Q., Liu C., Gao T., Gao L., Saxén H., Zevenhoven R. (2019). Remediation of stainless steel slag with MnO for CO_2_ mineralization. Process Saf. Environ. Prot..

[180] Telukdarie A., Buhulaiga E., Bag S., Gupta S., Luo Z. (2018). Industry 4.0 implementation for multinationals. Process Saf. Environ. Prot..

[181] Moktadir M.A., Ali S.M., Kusi-Sarpong S., Shaikh M.A.A. (2018). Assessing challenges for implementing Industry 4.0: Implications for process safety and environmental protection. Process Saf. Environ. Prot..

[182] Logan M., Safi M., Lens P., Visvanathan C. (2019). Investigating the performance of internet of things based anaerobic digestion of food waste. Process Saf. Environ. Prot..

[183] Lu Y., Liu Y., Shi S., Wang G.G.X., Li H., Wang T. (2020). Micro-particles stabilized aqueous foam for coal spontaneous combustion control and its flow characteristics. Process Saf. Environ. Prot..

[184] Liu W., Qin Y., Shi C., Guo D. (2019). Dynamic evolution of spontaneous combustion of coal in longwall gobs during mining-stopped period. Process Saf. Environ. Prot..

[185] Zhang Y., Zou Q., Guo L. (2020). Air-leakage Model and sealing technique with sealing–isolation integration for gas-drainage boreholes in coal mines. Process Saf. Environ. Prot..

[186] Liu T., Lin B., Fu X., Zhu C. (2020). Modeling air leakage around gas extraction boreholes in mining-disturbed coal seams. Process Saf. Environ. Prot..

[187] Zeng T., Chen G., Yang Y., Chen P., Reniers G. (2020). Developing an advanced dynamic risk analysis method for fire-related domino effects. Process Saf. Environ. Prot..

[188] Eslami Baladeh A., Cheraghi M., Khakzad N. (2019). A multi-objective model to optimal selection of safety measures in oil and gas facilities. Process Saf. Environ. Prot..

[189] Ghuge S.P., Saroha A.K. (2018). Catalytic ozonation of dye industry effluent using mesoporous bimetallic Ru-Cu/SBA-15 catalyst. Process Saf. Environ. Prot..

[190] Volpin F., Fons E., Chekli L., Kim J.E., Jang A., Shon H.K. (2018). Hybrid forward osmosis-reverse osmosis for wastewater reuse and seawater desalination: Understanding the optimal feed solution to minimise fouling. Process Saf. Environ. Prot..

[191] Sidik D.A.B., Hairom N.H.H., Ahmad M.K., Madon R.H., Mohammad A.W. (2020). Performance of membrane photocatalytic reactor incorporated with ZnO-Cymbopogon citratus in treating palm oil mill secondary effluent. Process Saf. Environ. Prot..

[192] Jia Z., Zeng W., Xu H., Li S., Peng Y. (2020). Adsorption removal and reuse of phosphate from wastewater using a novel adsorbent of lanthanum-modified platanus biochar. Process Saf. Environ. Prot..

[193] Dragoi E.N., Kovács Z., Juzsakova T., Curteanu S., Cretescu I. (2018). Environmental assesment of surface waters based on monitoring data and neuro-evolutive modelling. Process Saf. Environ. Prot..

[194] Zaranezhad A., Asilian Mahabadi H., Dehghani M.R. (2019). Development of prediction models for repair and maintenance-related accidents at oil refineries using artificial neural network, fuzzy system, genetic algorithm, and ant colony optimization algorithm. Process Saf. Environ. Prot..

[195] Li F., Hemmati A., Rashidi H. (2020). Industrial CO_2_ absorption into methyldiethanolamine/piperazine in place of monoethanolamine in the absorption column. Process Saf. Environ. Prot..

[196] Tong R., Cheng M., Yang X., Yang Y., Shi M. (2019). Exposure levels and health damage assessment of dust in a coal mine of Shanxi Province, China. Process Saf. Environ. Prot..

[197] Azimi S.C., Shirini F., Pendashteh A. (2019). Evaluation of COD and turbidity removal from woodchips wastewater using biologically sequenced batch reactor. Process Saf. Environ. Prot..

[198] Tolba A., Gar Alalm M., Elsamadony M., Mostafa A., Afify H., Dionysiou D.D. (2019). Modeling and optimization of heterogeneous Fenton-like and photo-Fenton processes using reusable Fe_3_O_4_-MWCNTs. Process Saf. Environ. Prot..

[199] Schmitz P., Swuste P., Reniers G., van Nunen K. (2020). Mechanical integrity of process installations: Barrier alarm management based on bowties. Process Saf. Environ. Prot..

[200] Li M., Wang D., Shan H. (2019). Risk assessment of mine ignition sources using fuzzy Bayesian network. Process Saf. Environ. Prot..

[201] Li M., Wang H., Wang D., Shao Z., He S. (2020). Risk assessment of gas explosion in coal mines based on fuzzy AHP and bayesian network. Process Saf. Environ. Prot..

[202] Abubakirov R., Yang M., Khakzad N. (2020). A risk-based approach to determination of optimal inspection intervals for buried oil pipelines. Process Saf. Environ. Prot..

